# A Network Analysis of Drug Combinations Associated with Acute Generalized Exanthematous Pustulosis (AGEP)

**DOI:** 10.3390/jcm10194486

**Published:** 2021-09-29

**Authors:** Adrian Martinez-De la Torre, Eva van Weenen, Mathias Kraus, Stefan Weiler, Stefan Feuerriegel, Andrea M. Burden

**Affiliations:** 1 Institute of Pharmaceutical Sciences, Department of Chemistry and Applied Biosciences, ETH Zurich, 8093 Zurich, Switzerland; adrian.martinez@pharma.ethz.ch (A.M.-D.l.T.); stefan.weiler@pharma.ethz.ch (S.W.); 2 Management Information Systems, Department of Management, Technology and Economics, ETH Zurich, 8092 Zurich, Switzerland; evanweenen@ethz.ch (E.v.W.); mathiaskraus@ethz.ch (M.K.); sfeuerriegel@ethz.ch (S.F.)

**Keywords:** acute generalized exanthematous pustulosis, AGEP, drug combination, network analysis

## Abstract

Acute generalized exanthematous pustulosis (AGEP) is a rare skin adverse drug reaction. The pathophysiology and causative drugs associated with AGEP are poorly understood, with the majority of studies in AGEP focusing on a single-drug-outcome association. We therefore aimed to explore and characterize frequently reported drug combinations associated with AGEP using the WHO pharmacovigilance database VigiBase. In this explorative cross-sectional study of a pharmacovigilance database using a data-driven approach, we assessed individual case safety reports (ICSR) with two or more drugs reported to VigiBase. A total of 2649 ICSRs reported two or more drugs. Cardiovascular drugs, including antithrombotics and beta-blockers, were frequently reported in combination with other drugs, particularly antibiotics. The drug pair of amoxicillin and furosemide was reported in 57 ICSRs (2.2%), with an O/E ratio of 1.3, and the combination of bisoprolol and furosemide was recorded 44 times (1.7%), with an O/E ratio of 5.5. The network analysis identified 10 different communities of varying sizes. The largest cluster primarily consisted of cardiovascular drugs. This data-driven and exploratory study provides the largest real-world assessment of drugs associated with AGEP to date. The results identify a high frequency of cardiovascular drugs, particularly used in combination with antibiotics.

## 1. Introduction

Acute generalized exanthematous pustulosis (AGEP) is a rare but severe skin adverse drug reaction (ADR) [1]. This reaction is described as a neutrophilic hypersensitivity reaction (type IVd) [2], and it is characterized by the sudden presence of several miniscule nonfollicular pustules over an edematous erythema [1,3]. In addition to the skin reaction, high fever and high neutrophil counts are frequent [4]. Fatal outcomes occur in <5% of patients [5], which are frequently due to secondary infections and complications triggering a fatal cascade of events [6,7].

While AGEP has occasionally been associated with the use of contrast agent dyes [8,9] and viruses [10], more than 90% of the cases are associated with drugs [4], with symptoms usually appearing within the first 24 h from intake [11]. To date, regulatory authorities have included warnings and watchlists for multiple individual drugs. For example, the European Medicines Agency (EMA) has included (or recommended) warnings for flucloxacillin [12], acetazolamide [13], cefalexin [14], and most recently, ibuprofen [15]. In the US, the Food and Drug Administration (FDA) added aripiprazole [16], vancomycin [17], hydroxyzine pamoate, levocetirizine, cetirizine, and 13 different proton-pump inhibitors to a watchlist for AGEP [18].

Following diagnosis, patch tests can elucidate the responsible drug. However, they do not assess the potential for drug-drug interactions or the additive effect of multiple medications taken concomitantly [19]. To date, the majority of studies focus on a single causative agent, and antibiotics are the most frequently identified cause of AGEP [11,20,21,22]. Moreover, common algorithms for signal detection in pharmacovigilance databases used by regulatory agencies, such as the EMA and FDA, only screen for one drug at a time [23].

While the concomitant use of multiple drugs (i.e., polypharmacy) is a known risk factor for ADRs, only a few studies have examined concomitant medication use and AGEP [11,24,25,26]. Within signal detection, advanced statistical models, such as network analysis, have previously been applied in pharmacovigilance databases to model drug-drug-disease combinations [27], adverse drug reactions to the H1N1 influenza vaccine [28], or for predicting adverse drug events [29]. These approaches have proven to be effective to drive new hypotheses regarding drug-outcome associations by capturing complex patterns and connections that would be difficult to detect and model otherwise. Thus, as the underlying causative agents of AGEP remain unknown, we employed an exploratory data-driven approach in order to identify frequently occurring drug combinations and drug clusters among patients with AGEP.

## 2. Materials and Methods

### 2.1. Data Source and Patient Population

We extracted all individual case safety reports (ICSRs) with AGEP (search terms “acute generalised exanthematous pustulosis”, “acute generalized exanthematous pustulosis”, and “AGEP” as low-level terms) recorded as the suspected ADR according to the Medical Dictionary for Regulatory Activities (MedDRA) Preferred Term version 22.1 from VigiBase via VigiLyze (https://vigilyze.who-umc.org/ (accessed on 14 January 2021)). We included data from database inception to 10 January 2021. VigiLyze is an online platform which enables the retrieval and visualization of data from VigiBase, the World Health Organization (WHO) global database of ICSRs [30]. VigiBase is managed by the Uppsala Monitoring Centre (UMC) and contains ICSRs from >130 countries, representing over 90% of the world’s population.

Among all ICSRs identified with AGEP recorded, we extracted all available information, including patient characteristics, reporter location and type, reported drugs and adverse events, seriousness, onset date, resolution, and death. Reported medications are identified by the WHODrug dictionary [30], which classifies drugs based on the active ingredient. All reported drugs are categorized as being either suspected, interacting, or concomitant for the reported adverse event. To facilitate grouping of drugs by drug class or therapeutic indication, we assigned the anatomical therapeutic chemical classification (ATC) code to the drugs. We excluded reported medications if the drug name was unknown or the medication could not be categorized by an ATC code. We further excluded ICSRs that were deemed by the UMC as possible duplicates. Finally, in order to assess combinations of medications, we restricted our analysis to those patients who took more than one drug.

### 2.2. Analysis

We summarized ICSR characteristics overall and stratified by sex and age categories. Means and standard deviations, or counts and proportions, were reported, and differences between strata were tested using the chi-squared test and the *t*-test, as appropriate. The top 20 reported drugs, and the most frequent pairs of drugs and drug triads were summarized. We further computed the observed to expected ratio (O/E ratio) for each drug pair or triad [31]. The O/E ratio measures if a specific combination appears more times than expected (Supplementary Text S1). O/E ratios larger than one imply that a specific pair or triad was more prevalent than expected.

To further explore drug combinations, we conducted a network analysis, which is an advanced statistical analysis that can characterize interconnected structures in data [32,33]. Network analyses comprise nodes and edges, where nodes represent the drugs and the edges between two nodes represent whether these two drugs were concomitantly taken by an individual patient. The size of a node is proportional to the number of times the drug was reported, and the width of an edge is proportional to the number of times that that specific connection occurs, representing its weight. All edges are bidirectional resulting in an undirected network. The node color corresponds to the anatomical main group (ATC-1) of the drug according to the ATC system.

Once the weighted and undirected network was constructed, we applied the Louvain algorithm, which is a multi-level modularity optimization algorithm, to find community structure and identify clusters where the nodes are highly interconnected [34]. This algorithm aims to maximize the modularity, which measures the density of connections within clusters compared to the density of connections between clusters. This is done by assigning each node its own cluster, then iteratively checking if adding the neighbors of a node to the same cluster increases the modularity. The process is continued until a stable solution is reached without an increase in modularity. Once the different clusters were obtained, we summarized the top 20 drugs with higher prevalence overall and reported the corresponding node degree (the number of connections it has to other nodes), the number of ICSRs in which each drug was reported, and its equivalent prevalence.

As a secondary analysis, we stratified the network analysis by sex (female vs. male) and age (<65 vs. >65 years old). As a robustness check, we applied a second clustering technique to the network analysis to test the robustness of our results. This approach aims to find densely connected subgroups by computing the leading non-negative eigenvector of the modularity matrix [35]. It calculates the eigenvector of the modularity matrix for the largest positive eigenvalue and then it separates its vertices into communities depending on the sign of the corresponding eigenvector. Data cleaning, modelling, summary tables, and O/E ratios were conducted in R [36], and network visualization in Gephi [37].

Data was accessed via the WHO VigiLyze signal detection and management system, which is available to member countries of the WHO Programme and healthcare professionals [30]. The extracted anonymized ICSR are those that have been routinely collected through the WHO Programme for International Drug Monitoring since 1968 as a public health service and for research purposes. All procedures and analyses adhered to the Uppsala Monitoring Centre (UMC) caveat agreement for reporting standards and in accordance with the Helsinki declaration for ethical principles in medical research. As the data was anonymized, and all analyses were descriptive, an ethical review from the Zurich Cantonal Ethics Board was not required. Additionally, because all data in VigiBase are anonymized, patient informed consent was also not required. All methods were carried out in accordance with the STROBE guidelines [38].

## 3. Results

### Descriptive Analysis

**Figure 1:**
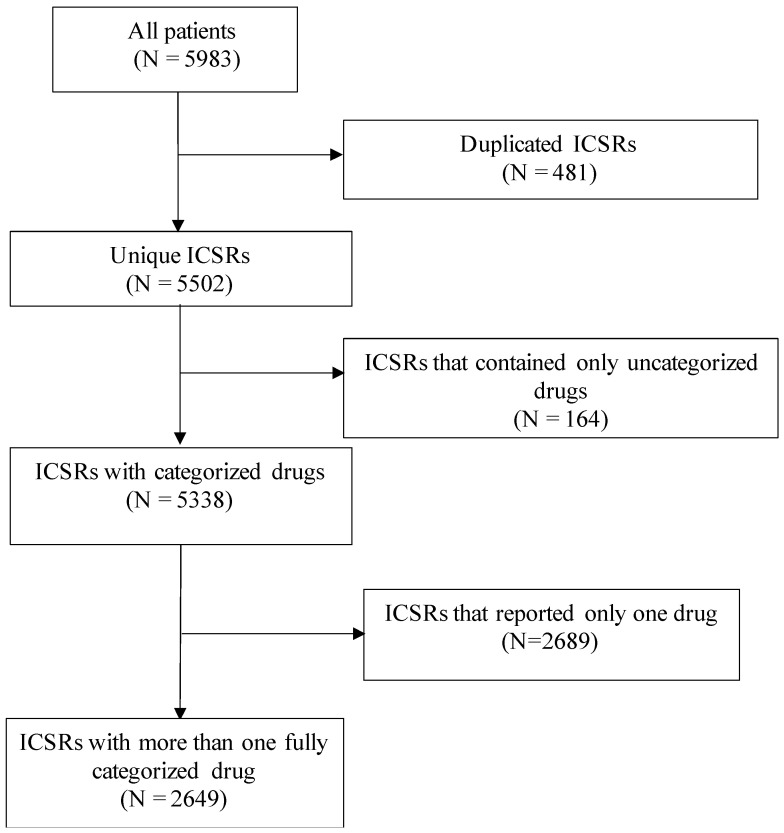
Flowchart of inclusion criteria. Acronyms: ICSR: individual case safety report.

We extracted 5983 ICSRs with AGEP reported to the WHO VigiBase. Following exclusions (Figure 1), 2649 ICSRs with two or more reported drugs were identified, of which 1571 (59.3%) were female, 1020 (38.5%) were male, and 58 (2.2%) had unknown sex (Table 1). Overall, the average age of the patients was 57.3 years. Most of the reports (59.8%) corresponded to cases in Europe. In 91.9% of the reports, AGEP diagnosis was categorized as a serious adverse reaction and 2.5% of the patients had a fatal outcome. When comparing females and males, we identified that females were generally older (59.3 years vs. 54.3 years, respectively) and had a slightly higher proportion of serious outcomes (92.2% vs. 91.2%, respectively). Conversely, the proportion with a fatal outcome was slightly higher among males than females (2.6% vs. 2.1%, respectively). When stratified by age (<65 vs. >65 years old), we observed that those aged over 65 years were more likely to have a serious outcome (94.7% vs. 89.1%, *p* < 0.001) (Supplementary Table S1). The older group presented 43 (65.2%) of the 66 fatal events.

Table 2 displays the reported drugs associated with AGEP among ICSRs with two or more drugs recorded in the database. The most frequently reported drug was amoxicillin, with an overall prevalence of 21.6%, followed by paracetamol (15.0%), ceftriaxone (8.8%), vancomycin (8.3%), and furosemide (7.4%). Cardiovascular drugs were reported in 894 of the 2649 (33.7%) ICSRs. The most prevalent cardiovascular drugs were furosemide (*n* = 197, 7.4%), acetylsalicylic acid (*n* = 189, 7.1%), amlodipine (*n* = 126, 4.8%), enoxaparin (*n* = 118, 4.5%), and bisoprolol (*n* = 108, 4.1%).

When assessing pairs of drugs (Table 2), we found that paracetamol and amoxicillin was the most commonly reported combination (*n* = 109, 4.1%) with an O/E ratio of 1.3, followed by amoxicillin and furosemide (*n* = 57, 2.2%) with an O/E ratio of 1.3. The highest O/E ratio from the top 20 drug-drug pairs was observed for the combination of levetiracetam and valproic acid (O/E ratio 20.6), followed by atorvastatin and acetylsalicylic acid (O/E ratio 6.5). Combinations of antibiotics and cardiovascular drugs had elevated O/E ratios. For example, the bisoprolol and furosemide was reported 44 times (1.7%) with an O/E ratio of 5.5. Similar combinations were observed when identifying drug triads (Table 2). The most frequently reported triad was ibuprofen, amoxicillin, and paracetamol (*n* = 17, 0.6%) with an O/E ratio of 5.1, followed by enoxaparin, amoxicillin, and paracetamol (*n* = 16, 0.6%) with an O/E ratio of 4.2, and acid acetylsalicylic, furosemide, and amoxicillin (*n* = 14, 0.6%) with an O/E ratio of 4.9.

A visual representation of the complete network analysis of drug combinations is presented in Supplementary Figure S1, where ten different clusters with varying sizes were identified. The main drugs of each cluster are shown in Supplementary Table S2. The largest cluster included 283 different drugs, where the largest proportion of these, 77 (27.2%), corresponded to cardiovascular drugs. The second and third largest clusters, with 212 and 97 drugs, primarily included nervous system drugs and antineoplastic and immunomodulating drugs, respectively.

**Figure 2:**
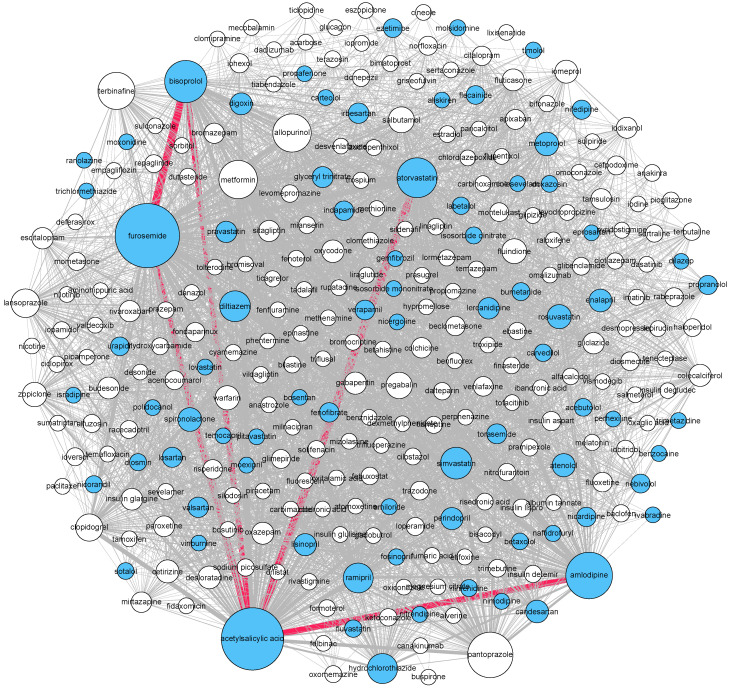
Cluster 2 (cardiovascular cluster) with cardiovascular drugs highlighted in blue, and most relevant edges highlighted in red. Nodes represent medications, the sizes of the nodes are proportional to the prevalence of the drug, the links indicate that the two connected drugs were taken concomitantly, the width of the link is proportional to the number of times the pair of drugs was reported.

**Figure 3:**
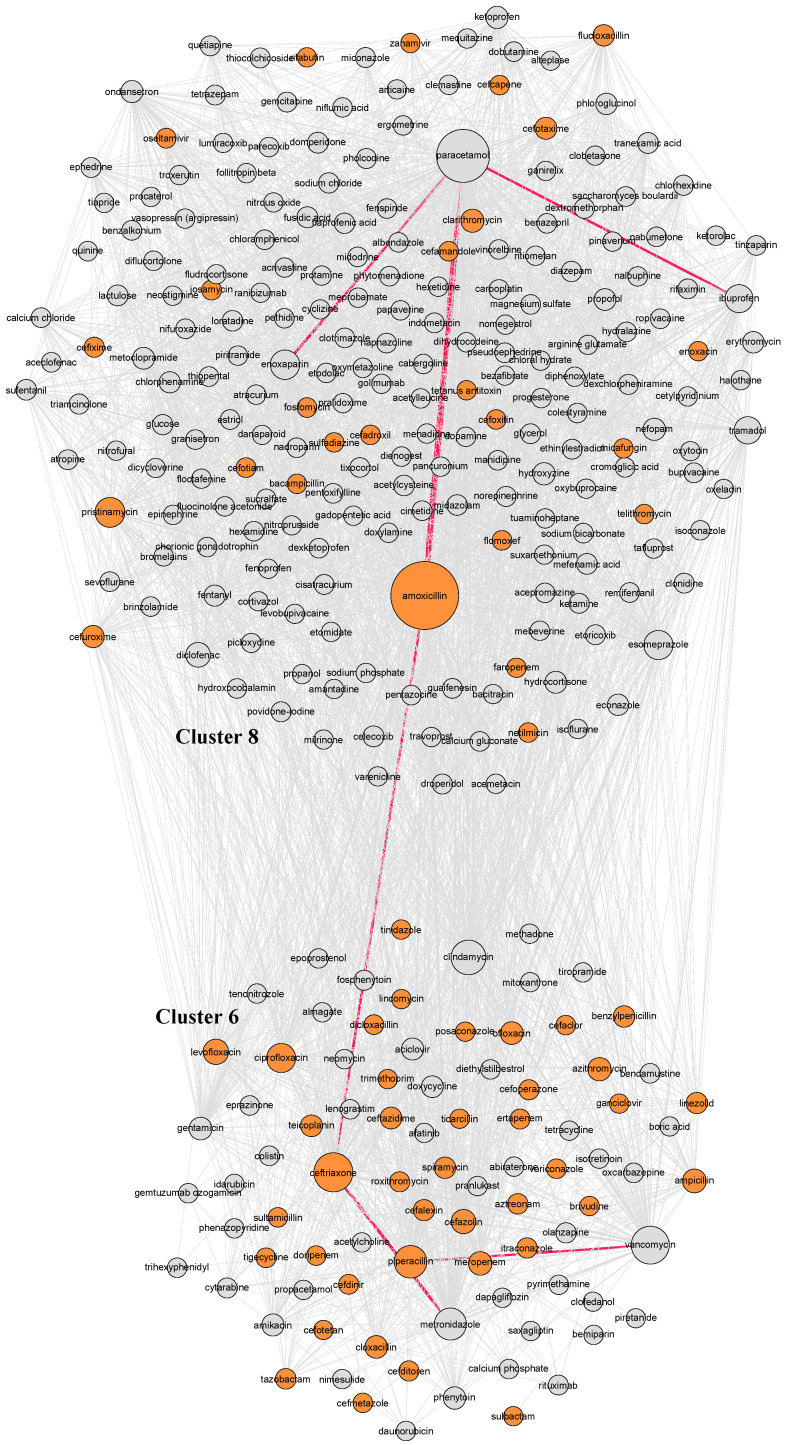
Clusters 6 and 8 (antimicrobial clusters) with antibiotic drugs highlighted in orange, and most relevant edges highlighted in red. Notes: Nodes represent medications, the sizes of the nodes are proportional to the prevalence of the drug, the links indicate that the two connected drugs were taken concomitantly, the width of the link is proportional to the number of times the pair of drugs was reported.

Figure 2 displays the network for the largest cluster, where the thickest edge was observed between acetylsalicylic acid and atorvastatin with a weight of 46 (i.e., 46 individuals took them both concomitantly), followed by furosemide and bisoprolol with a weight of 44. In the third largest cluster (Figure 3), composed of anti-infectives for systemic use, the thickest edge was between amoxicillin and paracetamol with a weight of 109.

The secondary analyses, stratified by sex and age, revealed similar results to the primary analysis (Supplementary Tables S3–S6). Additionally, the robustness check of clustering the network based on the leading eigenvalue revealed four distinct clusters as shown in Supplementary Table S7. These were similar to to those identified in the primary analysis, with Cluster 1 dominated by antibiotics (e.g., ceftriaxone, vancomycin, or clindamycin), while Cluster 3 had a high prevalence of cardiovascular drugs (e.g., furosemide, amlodipine, or bisoprolol).

## 4. Discussion

In this data-driven analysis of pharmacovigilance data among patients with AGEP and two or more reported medications, we identified frequent reporting of antibiotic and cardiovascular drugs. The reported use of both antibiotics and cardiovascular medications occurred with above-expected frequency and were identified with strong connections in both clustered network analyses. While we observed that females and older patients were more likely to experience serious outcomes, we did not identify any sex or age differences with drug combinations.

In line with the literature on AGEP-associated drug classes, we identified that antibiotics, such as amoxicillin, ceftriaxone, and clindamycin, were the most commonly reported, also in drug combinations. Overall, 62.1% of ICSRs included at least one antibiotic. Amoxicillin was the most frequently reported drug, followed by paracetamol, ceftriaxone, vancomycin, and clindamycin. A previous study by Barbaud and colleagues assessing the safety and value of drug patch testing, identified that 58% (26 of 45) of AGEP cases showed a positive result, of which 63% (16 of 26) were for pristinamycin or beta lactams [19]. Similarly, in the largest study investigating risk factors for AGEP to date, the EuroSCAR analysis, pristinamycin and ampicillin/amoxicillin were strongly associated with AGEP [11].

Additionally, we found frequent reporting of cardiovascular drugs in combination with other medications. Cardiovascular drugs such as antithrombotic agents (e.g., clopidogrel, heparin), calcium channel blockers (e.g., amlodipine, lercanidipine, felodipine), beta-blockers (e.g., bisoprolol, metoprolol), or lipid modifying agents (e.g., simvastatin, atorvastatin, ezetimibe) were reported in one-third of all patients. We note that the EuroSCAR analysis found that the single-agent use of beta-blockers, calcium channel blockers, acetylsalicylic acid, and angiotensin converting enzymes (ACE) inhibitors were not associated with the development of AGEP [11]. However, in the study by Barbaud and colleagues, the use of heparin (enoxaparin) was associated with a positive drug patch test, suggesting the potential for antithrombotic drugs to induce AGEP [19]. However, neither study considered the potential of these drugs, or drug classes, in combination with other agents.

Thus, our results further expand on the existing literature by investigating reported drug combinations in pharmacovigilance data. Our analysis identified 2649 ICSRs, providing the opportunity to assess a large number of potential drug combinations. To the best of our knowledge, only four studies have investigated the potential for two or more drugs to be associated with AGEP [11,24,25,26]. Of these, three were case reports [24,25,26] where two specific drugs were identified, and the study by Barbaud and colleagues, which found that 7 out of 45 cases had taken several drugs concomitantly [19]. In our analysis, we identified increased O/E ratios among ICSRs reporting cardiovascular drugs, especially when several cardiovascular drugs were reported. For example, furosemide, bisoprolol, and amoxicillin (O/E ratio = 8.6), warfarin, furosemide, and bisoprolol (O/E ratio = 94.2), or allopurinol, furosemide, and bisoprolol (O/E ratio = 35.9).

Additionally, the network analysis allowed us to have a comprehensive visualization of all the concomitant medications. On this account, the goal of network analysis is twofold: at a macro level, it is to find larger clusters of commonly reported medications in AGEP onset, and at a micro level, it is to look at specific drug combinations. The two clustering algorithms applied to the networks consistently found two main clusters across age and gender, one with antibiotics and another cluster with cardiovascular drugs. The former is in line with the literature, as antibiotics have commonly been associated with AGEP onset [11,39]. The latter, the cardiovascular cluster, highlights the association of these drugs with AGEP. Moreover, relevant combinations to treat certain pathologies were clustered together and showed a high degree of connection. For instance, drug combinations like ceftriaxone, metronidazole, and piperacillin, commonly used in pneumonia or intra-abdominal infections, or furosemide, amlodipine, and bisoprolol, used in heart failure patients, were consistently clustered together in the primary and secondary analyses as well as in the robustness check. The fact that the network and cluster analyses found well-known drug combinations associated with AGEP onset highlights the robustness of the approach used.

Conventional algorithms for signal detection in pharmacovigilance databases, such as the reporting odds ratio (ROR), information component (IC), or the empirical bayes geometric mean (EBGM), are limited due to the fact that only one drug and one ADR can be analyzed at a time. This can lead to misleading conclusions due to confounding and the inherent intertwined associations between drugs and adverse drug reactions that might be overlooked [23].

Network analysis has been used in pharmacovigilance data, as it can capture complex relationships between drugs and adverse drug reactions. For instance, Davazdahemami and colleagues used network analysis in combination with machine learning to predict adverse drug reactions [29]. Botsis and Ball used this approach to model vaccine adverse drug reactions [28,40], and Kim et al. applied network analysis to analyze the relationship between causative drugs and types of drug-related problems in patients with hematologic malignancies [41]. The complex pathophysiology of AGEP, the poor understanding of its etiology, and its low incidence, make network analysis a superior statistical approach compared to traditional approaches, especially to those used by the regulatory agencies. Moreover, by using a community detection algorithm, the Louvain algorithm, we managed to find novel and relevant combinations associated with AGEP onset.

To date, the pathophysiological mechanisms of AGEP have only been partially investigated, but some studies point at genetic or immunologic components as potential risk factors [42]. Immunologic cascades with cell-mediated inflammation, inflammatory cytokines, and chemokines can be triggered by infections or associated with cardiovascular diseases. Inflammatory cell accumulation and inflammatory cytokine production is interlinked with the initiation and progression of numerous cardiovascular diseases, such as myocardial infarction, remodeling, hypertension, or heart failure [43,44]. On the other hand, multimorbidity, such as cardiovascular diseases with hypertension, heart failure, or dyslipidemia, is associated with polypharmacy. Thus, co-prescribed drugs might serve as a covalently bound xenobiotic-cell protein adduct that acts as a hapten (i.e., independent of their respective effects but in combination with the specific host immune system). For example, a drug combination with a high prevalence in our study was furosemide, amlodipine, and bisoprolol, which is a classic prescription pattern for cardiovascular patients. Hence, it can be speculated that individuals with immune dysregulation or high polypharmacy burden might be exposed to an increased risk of potentially serious adverse reactions, such as AGEP.

The concept of drug interactions with AGEP is novel. However, there is growing evidence suggesting the potential for drug interactions to play a role in reactions with genetic and immunologic mechanisms, such AGEP, Stevens Johnson Syndrome (SJS), or toxic epidermal necrolysis (TEN) [43]. Several major histocompatibility complex haplotypes have been implicated in drug-specific susceptibilities for dermatological conditions, such as SJS, and a recent TENA study in the Japanese Adverse Drug Event Reporting (JADER) system identified drug combinations with antiepileptic drugs associated with SJS and TEN [44]. Another longitudinal cohort, using national Taiwanese insurance data, identified significant associations between the coadministration of drugs, such as allopurinol and ampicillin, or allopurinol and sulfamethoxazole, prior to fatal events among patients with SJS [45]. While these studies require further clinical validation, there is a growing interest in the potential for large datasets to provide new insights into these difficult, and potentially fatal, conditions.

### Limitations

We presented a data-driven cross-sectional analysis of pharmacovigilance data and, therefore, must be mindful of the intrinsic limitations when interpreting our results. VigiBase data is limited by the heterogeneity in reporting methodologies from the different regional and national pharmacovigilance centers [30]. While steps are taken to identify duplicates and improve data standardization, we are mindful that missing or incomplete data is an important shortcoming. For instance, we acknowledge the fact that we cannot assess how AGEP was diagnosed, e.g., patch testing or more drug-specific in vitro immunologic tests. In addition, given the nature of the database, we are unable to determine if the diagnosis of other conditions with similar presentation, such as pustular psoriasis, were ruled out prior to the diagnosis of AGEP. Indeed, in our study, 14 (5.3%) ICSRs had both AGEP and pustular psoriasis listed as possible outcomes. Indeed, when reporting a suspected ADR, only a single suspect drug is required. Thus, not all concomitantly used medications need to be identified in a report if they are not deemed to be relevant to the adverse event of interest. While our analysis restricted inclusion to reports where two or more drugs were reported, we cannot confirm that all concomitantly used drugs are accurately recorded, and we may have underestimated the true prevalence of drug combinations. We also did not limit our analysis based on the categorization of the individual medications (e.g., suspect, concomitant, or interacting). Future research with this type of data could consider additional weights for those drugs identified as suspect. However, we feel providing a broader analysis here enables further insights into potential interactions to be explored further.

We further note that, while the dates of the reported events and the drug start and stop can be reported, there was a high frequency of missingness in these fields. Consequently, we were unable to assess if medications were taken simultaneously, nor could we a determine temporal association between the medication and outcome. Thus, our results should be viewed as hypothesis-generating, as we did not aim to, nor could we, establish causality. Finally, while we stratified our analyses by age and sex, it is possible that our results are confounded by the occurrence of comorbidities that could not be adjusted for in this database. For example, an underlying cardiovascular disease could be associated with an AGEP onset directly or indirectly by increasing the risk of susceptibility of AGEP. In this case, the disease, rather than the concomitant drug, may be the underlying precipitating factor. It should also be noted that we did not identify any known drug-drug interactions based on pharmacokinetic or pharmacodynamics interactions in relation to AGEP. Thus, the observed combinations may be due to underlying confounding, or a previously unknown off-target drug binding effect. Therefore, we strongly encourage future work to expand on our results to both investigate the mechanism of action and the risk of AGEP associated with cardiovascular drugs, both alone and in combination with antibiotics.

## 5. Conclusions

In this analysis of global pharmacovigilance data among suspected cases of AGEP with two or more reported drugs, we identified that cardiovascular drugs were frequently reported in combination with other drugs, including antibiotics. Among the identified drug combinations, we did not identify any known on-target drug-drug interactions based on classical pharmacokinetic or pharmacodynamic processes. These results, therefore, open new hypotheses regarding the potential for off-target drug-drug interactions, or additive drug effects, in relation to the onset of AGEP. However, as this is the first study to our knowledge applying advanced statistical methods to assess drug combinations associated with AGEP onset in pharmacovigilance data, additional studies are needed to control for potential confounding and selection bias in order to confirm the patterns observed. We therefore encourage additional studies, using both pharmacovigilance and large observational cohorts, to further elucidate the drug combinations observed in our pharmacovigilance data.

## Figures and Tables

**Table 1:** Demographic characteristics of patients included in the network analysis, stratified by sex.

	Overall		Female		Male		
	(*n* = 2649)		(*n* = 1571)		(*n* = 1020)		
	*n*	%	*n*	%	*n*	%	*p*-Value
Mean age (SD)	57.32	(21.80)	59.33	(21.34)	54.27	(22.14)	<0.001
Age Groups							<0.001
<16	114	(4.3)	41	(2.6)	72	(7.1)	
16–44	542	(20.5)	327	(20.8)	213	(20.9)	
45–64	723	(27.3)	405	(25.8)	317	(31.1)	
65–84	853	(32.2)	538	(34.2)	308	(30.2)	
85+	190	(7.2)	144	(9.2)	46	(4.5)	
Unknown	227	(8.6)	116	(7.4)	64	(6.3)	
Region of Report							<0.001
Europe	1585	(59.8)	1005	(64.0)	563	(55.2)	
Asia	619	(23.4)	339	(21.6)	274	(26.9)	
Africa	26	(1.0)	10	(0.6)	16	(1.6)	
North America	374	(14.1)	190	(12.1)	151	(14.8)	
Oceania	35	(1.3)	19	(1.2)	14	(1.4)	
South America	10	(0.4)	8	(0.5)	2	(0.2)	
Reporter Type							0.037
Physician	1739	(83.3)	1067	(85.4)	642	(81.3)	
Other Health Professional	296	(14.2)	157	(12.6)	124	(15.7)	
Nonhealth Professional	53	(2.5)	25	(2.0)	24	(3.0)	
Seriousness (Yes)	2179	(91.9)	1315	(92.2)	813	(91.2)	0.452
Death	66	(2.5)	33	(2.1)	27	(2.6)	0.441
Number of reported Drugs							
Mean (SD)	4.22	(3.24)	4.27	(3.20)	4.15	(3.32)	0.361
Median (IQR)	3.00	(2.00– 5.00)	3.00	(2.00– 5.00)	3.00	(2.00–5.00)	0.359

Abbreviations: SD, standard deviation; IQR, interquartile range. *p*-values correspond to comparison of female vs. male. Chi-Square was used for categorical variables, while *t*-test for numerical variables. There were 58 ICSRs where sex was unknown, therefore the total number of females and males does not sum to the overall.

**Table 2:** Most frequent drugs for the 2649 ICSRs included in the network analysis.

Individual Drugs			Drug-Drug Pairs						Drug Triads						
Drug 1	*n*	Prevalence	Drug 1	Drug 2	*n*	Prevalence	Expected Prevalence	O/E Ratio	Drug 1	Drug 2	Drug 3	*n*	Prevalence	Expected Prevalence	O/E Ratio
amoxicillin	573	21.63%	paracetamol	amoxicillin	109	4.11%	3.24%	1.27	ibuprofen	amoxicillin	paracetamol	17	0.64%	0.12%	5.14
paracetamol	397	14.99%	amoxicillin	furosemide	57	2.15%	1.61%	1.34	enoxaparin	amoxicillin	paracetamol	16	0.60%	0.14%	4.18
ceftriaxone	234	8.83%	ceftriaxone	metronidazole	53	2.00%	0.50%	4.00	ASA	furosemide	amoxicillin	15	0.57%	0.11%	4.93
vancomycin	219	8.27%	paracetamol	enoxaparin	47	1.77%	0.67%	2.66	furosemide	bisoprolol	amoxicillin	15	0.57%	0.07%	8.63
furosemide	197	7.44%	atorvastatin	ASA	46	1.74%	0.27%	6.51	ASA	amoxicillin	paracetamol	14	0.53%	0.23%	2.28
ASA	189	7.13%	amoxicillin	ASA	45	1.70%	1.54%	1.10	ASA	metformin	amlodipine	13	0.49%	0.01%	41.64
clindamycin	181	6.83%	ceftriaxone	amoxicillin	45	1.70%	1.91%	0.89	ASA	clopidogrel	atorvastatin	13	0.49%	0.00%	103.73
piperacillin	157	5.93%	bisoprolol	furosemide	44	1.66%	0.30%	5.48	ASA	amlodipine	atorvastatin	12	0.45%	0.01%	35.72
metronidazole	150	5.66%	paracetamol	omeprazole	42	1.59%	0.78%	2.05	ASA	furosemide	bisoprolol	11	0.42%	0.02%	19.20
omeprazole	137	5.17%	paracetamol	ibuprofen	41	1.55%	0.58%	2.68	metronidazole	vancomycin	ceftriaxone	11	0.42%	0.04%	10.04
amlodipine	126	4.76%	paracetamol	furosemide	41	1.55%	1.11%	1.39	warfarin	furosemide	bisoprolol	10	0.38%	0.00%	94.23
pristinamycin	124	4.68%	piperacillin	vancomycin	40	1.51%	0.49%	3.08	ASA	furosemide	atorvastatin	10	0.38%	0.02%	19.04
pantoprazole	121	4.57%	furosemide	ASA	39	1.47%	0.53%	2.77	ASA	atorvastatin	amoxicillin	10	0.38%	0.06%	6.55
prednisolone	120	4.53%	levetiracetam	valproic acid	37	1.40%	0.07%	20.60	warfarin	furosemide	amoxicillin	10	0.38%	0.02%	17.76
enoxaparin	118	4.45%	ceftriaxone	vancomycin	36	1.36%	0.73%	1.86	furosemide	amoxicillin	allopurinol	10	0.38%	0.06%	6.76
esomeprazole	116	4.38%	amlodipine	ASA	35	1.32%	0.34%	3.89	furosemide	bisoprolol	allopurinol	10	0.38%	0.01%	35.85
ciprofloxacin	116	4.38%	clindamycin	vancomycin	35	1.32%	0.56%	2.34	esomeprazole	enoxaparin	paracetamol	10	0.38%	0.03%	12.91
bisoprolol	108	4.08%	amoxicillin	ibuprofen	35	1.32%	0.83%	1.59	ASA	furosemide	paracetamol	10	0.38%	0.08%	4.75
sulfamethoxazole	106	4.00%	paracetamol	ASA	34	1.28%	1.07%	1.20	amoxicillin	ceftriaxone	paracetamol	10	0.38%	0.29%	1.32
ibuprofen	102	3.85%	bisoprolol	ASA	33	1.25%	0.29%	4.28	furosemide	amoxicillin	paracetamol	10	0.38%	0.24%	1.57

Abbreviations: O/E = observed to expected; ASA: acetylsalicylic acid.

## Data Availability

All methods were carried out in accordance with the STROBE guidelines [44]. The datasets used and/or analyzed during the current study are not publicly available, but the code and the anonymized dataset can be made available from the corresponding author upon reasonable request.

## Supplementary Materials

The following are available online at https://www.mdpi.com/article/10.3390/jcm10194486/s1, Supplementary Text S1: Observed to expected ratio calculation. Supplementary Figure S1: network overview of 2649 ICSRs that reported >2 drugs after applying the Louvain algorithm for cluster detection. In total, ten different clusters were identified. Nodes represent medications, the size of the nodes are proportional to the prevalence of the drug, the links indicate that the two connected drugs were taken concomitantly, the width of the link is proportional to the number of times the pair of drugs was reported. Only labels of nodes with a degree higher than 75 are shown. Supplementary Table S1: Demographic characteristics of individual case safety reports with AGEP, stratified by age. Supplementary Table S2: Summary of the top 20 drugs of each cluster found with the Louvain algorithm in the network analysis (*n* = 2649). Supplementary Table S3: Summary of the top 20 drugs of each cluster found with the Louvain algorithm in the network analysis among male ICSRs (*n* = 1020). Supplementary Table S4: Summary of the top 20 drugs of each cluster found with the Louvain algorithm in the network analysis among female ICSRs (*n* = 1571). Supplementary Table S5: Summary of the top 20 drugs of each cluster found with the Louvain algorithm in the network analysis among ICSRs aged < 65 years old (*n* = 1379). Supplementary Table S6: Summary of the top 20 drugs of each cluster found with the Louvain algorithm in the network analysis among ICSRs aged >65 years old (*n* = 1043). Supplementary Table S7: Summary of the top 20 drugs of each cluster found by using the leading eigenvector clustering to the network analysis (*n* = 2649).
