# Supplementary material for: A Network Analysis of Drug Combinations Associated with Acute Generalized Exanthematous Pustulosis (AGEP)
Source: J Clin Med. 2021 Sep 29;10(19):4486. doi: 10.3390/jcm10194486 (PMC8509508; doi:10.3390/jcm10194486)
Supplement: Supplementary file 1 [file jcm-10-04486-s001.zip › jcm-1374768-supplementary.pdf]

**Supplementary Material**

**Table of Contents**

**Supplementary Text S1.** Observed to Expected ratio calculation.

**Supplementary Figure S1** Network overview of 2649 ICSRs that reported ≥2 drugs after applying the Louvain algorithm for cluster detection. In total, ten different clusters were identified. Nodes represent medications, the size of the nodes are proportional to the prevalence of the drug, the links indicate that the two connected drugs were taken concomitantly, the width of the link is proportional to the number of times the pair of drugs was reported. Only labels of nodes with a degree higher than 75 are shown.

**Supplementary Table S1.** Demographic characteristics of individual case safety reports with AGEP, stratified by age.

**Supplementary Table S2.** Summary of the top 20 drugs of each cluster found with the Louvain algorithm in the network analysis (N = 2649).

**Supplementary Table S3.** Summary of the top 20 drugs of each cluster found with the Louvain algorithm in the network analysis among **male** ICSRs (N = 1020).

**Supplementary Table S4.** Summary of the top 20 drugs of each cluster found with the Louvain algorithm in the network analysis among **female** ICSRs (N = 1571).

**Supplementary Table S5.** Summary of the top 20 drugs of each cluster found with the Louvain algorithm in the network analysis among ICSRs aged **< 65 years old** (N = 1379).

**Supplementary Table S6.** Summary of the top 20 drugs of each cluster found with the Louvain algorithm in the network analysis among ICSRs aged **≥ 65 years old** (N = 1043).

**Supplementary Table S7.** Summary of the top 20 drugs of each cluster found by using the leading eigenvector clustering to the network analysis (N = 2649).

**Supplementary Text S1.** Observed to Expected ratio calculation.

Given a set of patients $\mathcal{P} = \{p_1, \dots, p_N\}$ and a set of medications $\mathcal{D} = \{d_1, \dots, d_M\}$, each patient $p_i \in \mathcal{P}$ is prescribed with a subset of medications $D_i \subseteq \mathcal{D}$. Therefore, our data is of the form $\{p_i, D_i\}_{i=1}^{N}$.

**Definition (Prevalence).** The prevalence of a drug $d_m \in \mathcal{D}$ is defined as

$$P(d_m) = \frac{1}{N}\sum_{i=1}^{N} \mathbb{1}_{D_i}(d_m),$$

where $\mathbb{1}$ is the *indicator function* $\mathbb{1}_A(x) = \begin{cases} 1 \text{ if } x \in A \\ 0 \text{ if } x \notin A \end{cases}$, indicating whether drug $d_m$ is in the medications $D_i$ of patient $p_i$.

The prevalence of a set of drugs $\mathcal{X} \subseteq \mathcal{D}$ taken together is then

$$P(\mathcal{X}) = \frac{1}{N}\sum_{i=1}^{N} \prod_{d_m \in \mathcal{X}} \mathbb{1}_{D_i}(d_m)$$

**Definition (O/E ratio).** The Observed to Expected ratio (O/E ratio) of a set of drugs $\mathcal{X} \subseteq \mathcal{D}$ is defined as the prevalence of the drugs in $\mathcal{X}$ taken together divided by the product of the prevalence of the individual drugs in $\mathcal{X}$.

$$\text{O/E ratio } (\mathcal{X}) = \frac{P(\mathcal{X})}{\prod_{d_m \in \mathcal{X}} P(d_m)} = \frac{\sum_{i=1}^{N} \prod_{d_m \in \mathcal{X}} \mathbb{1}_{D_i}(d_m)}{\prod_{d_m \in \mathcal{X}} \sum_{i=1}^{N} \mathbb{1}_{D_i}(d_m)}$$

**Example (O/E ratio).**

| Drug 1 | Drug 2 | Observed Prevalence | Prevalence Drug 1 | Prevalence Drug 2 | Expected Prevalence | O/E ratio |
|---|---|---|---|---|---|---|
| paracetamol | amoxicillin | 4.11% | 15.0% | 21.6% | 15.0% · 21.6% = 3.2% | $\frac{4.11\%}{3.2\%} = 1.28$ |

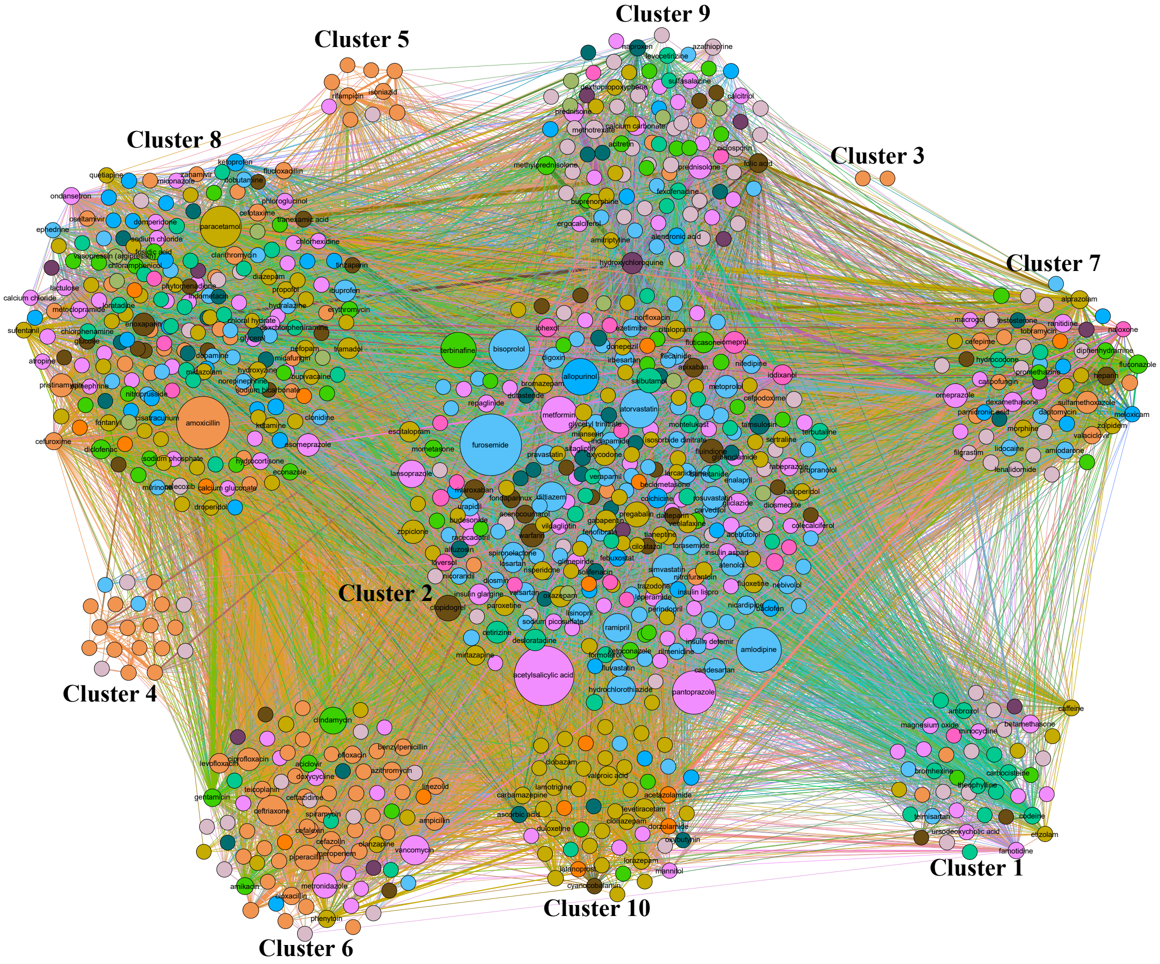


**Supplementary Figure S1** Network overview of 2649 ICSRs that reported ≥2 drugs after applying the Louvain algorithm for cluster detection. In total ten different clusters were identified. Nodes represent medications, the size of the nodes are proportional to the prevalence of the drug, the links indicate that the two connected drugs were taken concomitantly, the width of the link is proportional to the number of times the pair of drugs was reported. Only labels of nodes with a degree higher than 75 are shown.

**Supplementary Table S1.** Demographic characteristics of individual case safety reports with AGEP, stratified by age.

| | <65 (n=1379) | | ≥65 (n=1043) | | Unknown (n=227) | | |
|---|---|---|---|---|---|---|---|
| | N | % | N | % | N | % | p-value |
| **Sex** | | | | | | | |
| Male | 602 | (43.8) | 354 | (34.2) | 64 | (35.6) | <0.001 |
| **Region of Report** | | | | | | | <0.001 |
| Europe | 727 | (52.7) | 712 | (68.3) | 146 | (64.3) | |
| Asia | 435 | (31.5) | 161 | (15.4) | 23 | (10.1) | |
| Africa | 21 | (1.5) | 2 | (0.2) | 3 | (1.3) | |
| North America | 164 | (11.9) | 158 | (15.1) | 52 | (22.9) | |
| Oceania | 25 | (1.8) | 8 | (0.8) | 2 | (0.9) | |
| South America | 7 | (0.5) | 2 | (0.2) | 1 | (0.4) | |
| **Reporter Type** | | | | | | | 0.006 |
| Physician | 862 | (81.5) | 729 | (86.2) | 148 | (80.4) | |
| Other Health Professional | 167 | (15.8) | 103 | (12.2) | 26 | (14.1) | |
| Non Health Professional | 29 | (2.7) | 14 | (1.7) | 10 | (5.4) | |
| **Seriousness (Yes)** | 1058 | (89.1) | 920 | (94.7) | 201 | (94.8) | <0.001 |
| **Death** | 11 | (0.8) | 43 | (4.1) | 12 | (5.3) | <0.001 |
| **Number of reported Drugs** | | | | | | | |
| Mean (SD) | 3.68 | (2.65) | 4.86 | (3.66) | 4.53 | (3.80) | <0.001 |
| Median (IQR) | 3.00 | [2.00,4.00] | 3.00 | [2.00,6.00] | 3.00 | [2.00,6.00] | <0.001 |

Abbreviations: SD, Standard Deviation; IQR, interquartile range.

P-values correspond to comparison between younger (aged <65 years) and older (≥65 years) ICSRs

**Supplementary Table S2.** Summary of the top 20 drugs based on the reported drug for each of the clusters identified with the Louvain algorithm in the network analysis (N=2649).
Cardiovascular drugs highlighted in grey.

| **Cluster 1** ($N_{drugs}$=44) | | | | **Cluster 2** ($N_{drugs}$=283) | | | | **Cluster 3** ($N_{drugs}$=2) | | | |
|---|---|---|---|---|---|---|---|---|---|---|---|
| Drug | Degree | Count | Prevalence | Drug | Degree | Count | Prevalence | Drug | Degree | Count | Prevalence |
| betamethasone | 280 | 45 | 1.70% | furosemide | 688 | 197 | 7.44% | cefprozil | 2 | 1 | 0.04% |
| codeine | 248 | 26 | 0.98% | acetylsalicylic acid | 692 | 189 | 7.13% | famciclovir | 2 | 1 | 0.04% |
| carbocisteine | 196 | 23 | 0.87% | amlodipine | 534 | 126 | 4.76% | | | | |
| famotidine | 212 | 22 | 0.83% | pantoprazole | 538 | 121 | 4.57% | | | | |
| ambroxol | 180 | 17 | 0.64% | bisoprolol | 466 | 108 | 4.08% | | | | |
| caffeine | 154 | 16 | 0.60% | atorvastatin | 498 | 99 | 3.74% | | | | |
| magnesium oxide | 188 | 14 | 0.53% | metformin | 464 | 92 | 3.47% | | | | |
| bromhexine | 114 | 12 | 0.45% | allopurinol | 450 | 92 | 3.47% | | | | |
| minocycline | 78 | 11 | 0.42% | terbinafine | 294 | 87 | 3.28% | | | | |
| telmisartan | 104 | 9 | 0.34% | simvastatin | 406 | 65 | 2.45% | | | | |
| theophylline | 112 | 9 | 0.34% | diltiazem | 306 | 62 | 2.34% | | | | |
| ursodeoxycholic acid | 88 | 8 | 0.30% | hydrochlorothiazide | 354 | 59 | 2.23% | | | | |
| fluorouracil | 74 | 8 | 0.30% | ramipril | 324 | 58 | 2.19% | | | | |
| etizolam | 118 | 7 | 0.26% | lansoprazole | 328 | 53 | 2.00% | | | | |
| pemetrexed | 56 | 6 | 0.23% | clopidogrel | 330 | 47 | 1.77% | | | | |
| oxaliplatin | 34 | 6 | 0.23% | pregabalin | 358 | 44 | 1.66% | | | | |
| tipepidine | 38 | 5 | 0.19% | salbutamol | 338 | 42 | 1.59% | | | | |
| olopatadine | 48 | 4 | 0.15% | fluindione | 208 | 38 | 1.43% | | | | |
| tulobuterol | 46 | 4 | 0.15% | zopiclone | 326 | 36 | 1.36% | | | | |
| cetuximab | 16 | 3 | 0.11% | warfarin | 248 | 35 | 1.32% | | | | |

**Supplementary Table S2. (continued)** Summary of the top 20 drugs based on the reported drug for each of the clusters identified with the Louvain algorithm in the network analysis (N=2649).
Cardiovascular drugs highlighted in grey.

| **Cluster 4** ($N_{drugs}$=18) | | | | **Cluster 5** ($N_{drugs}$=10) | | | | **Cluster 6** ($N_{drugs}$=85) | | | |
|---|---|---|---|---|---|---|---|---|---|---|---|
| Drug | Degree | Count | Prevalence | Drug | Degree | Count | Prevalence | Drug | Degree | Count | Prevalence |
| ritonavir | 72 | 10 | 0.38% | rifampicin | 206 | 34 | 1.28% | ceftriaxone | 472 | 234 | 8.83% |
| darunavir | 68 | 9 | 0.34% | isoniazid | 92 | 19 | 0.72% | vancomycin | 392 | 219 | 8.27% |
| emtricitabine | 32 | 7 | 0.26% | pyrazinamide | 24 | 10 | 0.38% | clindamycin | 468 | 181 | 6.83% |
| lamivudine | 30 | 6 | 0.23% | moxifloxacin | 70 | 9 | 0.34% | piperacillin | 428 | 157 | 5.93% |
| cisplatin | 50 | 6 | 0.23% | ethambutol | 48 | 8 | 0.30% | metronidazole | 448 | 150 | 5.66% |
| etoposide | 58 | 4 | 0.15% | streptomycin | 14 | 1 | 0.04% | ciprofloxacin | 358 | 116 | 4.38% |
| bleomycin | 18 | 4 | 0.15% | cycloserine | 14 | 1 | 0.04% | levofloxacin | 300 | 72 | 2.72% |
| peginterferon alfa-2a | 6 | 4 | 0.15% | protionamide | 4 | 1 | 0.04% | cefazolin | 282 | 60 | 2.27% |
| nevirapine | 6 | 3 | 0.11% | ethionamide | 14 | 1 | 0.04% | ampicillin | 170 | 53 | 2.00% |
| efavirenz | 18 | 3 | 0.11% | tegafur | 8 | 1 | 0.04% | meropenem | 260 | 49 | 1.85% |
| atazanavir | 16 | 2 | 0.08% | | | | | azithromycin | 208 | 48 | 1.81% |
| didanosine | 18 | 2 | 0.08% | | | | | gentamicin | 264 | 42 | 1.59% |
| stavudine | 12 | 2 | 0.08% | | | | | cloxacillin | 162 | 40 | 1.51% |
| nelfinavir | 8 | 1 | 0.04% | | | | | ofloxacin | 216 | 40 | 1.51% |
| abacavir | 6 | 1 | 0.04% | | | | | phenytoin | 124 | 35 | 1.32% |
| raltegravir | 12 | 1 | 0.04% | | | | | amikacin | 180 | 34 | 1.28% |
| nadolol | 6 | 1 | 0.04% | | | | | aciclovir | 184 | 33 | 1.25% |
| zofenopril | 20 | 1 | 0.04% | | | | | doxycycline | 98 | 30 | 1.13% |
| | | | | | | | | ceftazidime | 118 | 29 | 1.09% |
| | | | | | | | | teicoplanin | 140 | 29 | 1.09% |

1

**Supplementary Table S2. (continued)** Summary of the top 20 drugs based on the reported drug for each of the clusters identified with the Louvain algorithm in the network analysis (N=2649). Cardiovascular drugs highlighted in grey.

| Cluster 7 ($N_{drugs}$=61) | | | | Cluster 8 ($N_{drugs}$=212) | | | |
|---|---|---|---|---|---|---|---|
| Drug | Degree | Count | Prevalence | Drug | Degree | Count | Prevalence |
| omeprazole | 614 | 137 | 5.17% | amoxicillin | 786 | 573 | 21.63% |
| sulfamethoxazole | 422 | 106 | 4.00% | paracetamol | 916 | 397 | 14.99% |
| dexamethasone | 366 | 61 | 2.30% | pristinamycin | 370 | 124 | 4.68% |
| fluconazole | 308 | 58 | 2.19% | enoxaparin | 484 | 118 | 4.45% |
| ranitidine | 330 | 47 | 1.77% | esomeprazole | 566 | 116 | 4.38% |
| heparin | 412 | 47 | 1.77% | ibuprofen | 324 | 102 | 3.85% |
| alprazolam | 398 | 46 | 1.74% | tramadol | 380 | 93 | 3.51% |
| cefepime | 164 | 39 | 1.47% | diclofenac | 296 | 64 | 2.42% |
| valaciclovir | 176 | 38 | 1.43% | clarithromycin | 228 | 45 | 1.70% |
| amiodarone | 244 | 32 | 1.21% | hydroxyzine | 256 | 36 | 1.36% |
| morphine | 282 | 31 | 1.17% | cefuroxime | 250 | 34 | 1.28% |
| diphenhydramine | 234 | 22 | 0.83% | nefopam | 252 | 34 | 1.28% |
| daptomycin | 148 | 21 | 0.79% | ketoprofen | 180 | 33 | 1.25% |
| lidocaine | 226 | 20 | 0.76% | ondansetron | 312 | 32 | 1.21% |
| zolpidem | 248 | 18 | 0.68% | cefotaxime | 216 | 32 | 1.21% |
| phenoxymethylpenicillin | 38 | 12 | 0.45% | metoclopramide | 260 | 31 | 1.17% |
| mupirocin | 22 | 12 | 0.45% | hydrocortisone | 334 | 30 | 1.13% |
| tobramycin | 188 | 11 | 0.42% | propofol | 242 | 28 | 1.06% |
| meloxicam | 100 | 11 | 0.42% | flucloxacillin | 182 | 26 | 0.98% |
| lenalidomide | 128 | 10 | 0.38% | erythromycin | 136 | 25 | 0.94% |

**Supplementary Table S2. (continued)** Summary of the top 20 drugs based on the reported drug for each of the clusters identified with the Louvain algorithm in the network analysis (N=2649). Cardiovascular drugs highlighted in grey.

| Cluster 9 ($N_{drugs}$=97) | | | | Cluster 10 ($N_{drugs}$=51) | | | |
|---|---|---|---|---|---|---|---|
| Drug | Degree | Count | Prevalence | Drug | Degree | Count | Prevalence |
| prednisolone | 432 | 120 | 4.53% | levetiracetam | 270 | 71 | 2.68% |
| hydroxychloroquine | 286 | 98 | 3.70% | valproic acid | 254 | 67 | 2.53% |
| prednisone | 356 | 76 | 2.87% | lorazepam | 262 | 28 | 1.06% |
| calcium carbonate | 472 | 62 | 2.34% | carbamazepine | 136 | 26 | 0.98% |
| folic acid | 404 | 61 | 2.30% | ascorbic acid | 232 | 24 | 0.91% |
| methylprednisolone | 264 | 51 | 1.93% | acetazolamide | 164 | 22 | 0.83% |
| methotrexate | 222 | 50 | 1.89% | lamotrigine | 166 | 19 | 0.72% |
| alendronic acid | 186 | 25 | 0.94% | duloxetine | 144 | 16 | 0.60% |
| azathioprine | 80 | 23 | 0.87% | clonazepam | 120 | 13 | 0.49% |
| naproxen | 120 | 23 | 0.87% | bupropion | 58 | 13 | 0.49% |
| ciclosporin | 128 | 17 | 0.64% | cyanocobalamin | 138 | 11 | 0.42% |
| fexofenadine | 156 | 17 | 0.64% | aripiprazole | 64 | 11 | 0.42% |
| levocetirizine | 140 | 15 | 0.57% | oxybutynin | 100 | 7 | 0.26% |
| acitretin | 114 | 14 | 0.53% | phenobarbital | 36 | 7 | 0.26% |
| amitriptyline | 134 | 14 | 0.53% | dorzolamide | 84 | 7 | 0.26% |
| sulfasalazine | 84 | 12 | 0.45% | latanoprost | 96 | 7 | 0.26% |
| calcitriol | 118 | 12 | 0.45% | clobazam | 76 | 6 | 0.23% |
| infliximab | 46 | 12 | 0.45% | mannitol | 110 | 5 | 0.19% |
| dextropropoxyphene | 94 | 12 | 0.45% | glucosamine | 34 | 5 | 0.19% |
| adalimumab | 52 | 9 | 0.34% | tropatepine | 68 | 5 | 0.19% |

**Supplementary Table S3.** Summary of the top 20 drugs of each cluster found with the Louvain algorithm in the network analysis among male ICSRs (N=1020).

| **Cluster 1** ($N_{drugs}$=193) | | | | **Cluster 2** ($N_{drugs}$=26) | | | | **Cluster 3** ($N_{drugs}$=104) | | | |
|---|---|---|---|---|---|---|---|---|---|---|---|
| Drug | Degree | Count | Prevalence | Drug | Degree | Count | Prevalence | Drug | Degree | Count | Prevalence |
| acetylsalicylic acid | 422 | 79 | 7.75% | carbocisteine | 96 | 12 | 1.18% | amoxicillin | 482 | 213 | 20.88% |
| furosemide | 366 | 68 | 6.67% | ambroxol | 102 | 9 | 0.88% | paracetamol | 568 | 167 | 16.37% |
| amlodipine | 288 | 47 | 4.61% | pemetrexed | 52 | 5 | 0.49% | ibuprofen | 184 | 49 | 4.80% |
| bisoprolol | 302 | 46 | 4.51% | caffeine | 48 | 5 | 0.49% | diclofenac | 140 | 29 | 2.84% |
| allopurinol | 282 | 44 | 4.31% | theophylline | 74 | 5 | 0.49% | tramadol | 144 | 27 | 2.65% |
| prednisolone | 244 | 41 | 4.02% | cefotiam | 14 | 3 | 0.29% | ampicillin | 80 | 23 | 2.25% |
| atorvastatin | 272 | 41 | 4.02% | etizolam | 70 | 3 | 0.29% | cloxacillin | 82 | 21 | 2.06% |
| pristinamycin | 174 | 40 | 3.92% | bromhexine | 48 | 3 | 0.29% | betamethasone | 102 | 17 | 1.67% |
| pantoprazole | 266 | 38 | 3.73% | tipepidine | 24 | 3 | 0.29% | benzylpenicillin | 40 | 16 | 1.57% |
| metformin | 208 | 30 | 2.94% | cefmetazole | 12 | 2 | 0.20% | chlorphenamine | 112 | 14 | 1.37% |
| cefazolin | 100 | 25 | 2.45% | telmisartan | 46 | 2 | 0.20% | cetirizine | 108 | 10 | 0.98% |
| ramipril | 172 | 25 | 2.45% | fenspiride | 10 | 2 | 0.20% | desloratadine | 112 | 10 | 0.98% |
| terbinafine | 106 | 24 | 2.35% | palonosetron | 34 | 1 | 0.10% | domperidone | 70 | 9 | 0.88% |
| levofloxacin | 160 | 23 | 2.25% | potassium citrate | 34 | 1 | 0.10% | celecoxib | 38 | 8 | 0.78% |
| simvastatin | 224 | 23 | 2.25% | cefozopran | 4 | 1 | 0.10% | cefixime | 18 | 7 | 0.69% |
| diltiazem | 130 | 22 | 2.16% | sitafloxacin | 8 | 1 | 0.10% | fusidic acid | 50 | 7 | 0.69% |
| folic acid | 170 | 19 | 1.86% | gefitinib | 34 | 1 | 0.10% | naproxen | 36 | 7 | 0.69% |
| lansoprazole | 146 | 18 | 1.76% | ticlopidine | 6 | 1 | 0.10% | ketoprofen | 32 | 7 | 0.69% |
| rosuvastatin | 112 | 16 | 1.57% | beraprost | 10 | 1 | 0.10% | mefenamic acid | 26 | 7 | 0.69% |
| iomeprol | 82 | 15 | 1.47% | carbazochrome | 34 | 1 | 0.10% | phloroglucinol | 56 | 6 | 0.59% |

**Supplementary Table S3. (continued)** Summary of the top 20 drugs of each cluster found with the Louvain algorithm in the network analysis among male ICSRs (N=1020).

| **Cluster 4** ($N_{drugs}$=2) | | | | **Cluster 5** ($N_{drugs}$=111) | | | | **Cluster 6** ($N_{drugs}$=11) | | | |
|---|---|---|---|---|---|---|---|---|---|---|---|
| Drug | Degree | Count | Prevalence | Drug | Degree | Count | Prevalence | Drug | Degree | Count | Prevalence |
| flomoxef | 2 | 1 | 0.10% | vancomycin | 268 | 87 | 8.53% | rifampicin | 160 | 17 | 1.67% |
| cefcapene | 2 | 1 | 0.10% | omeprazole | 376 | 59 | 5.78% | methotrexate | 74 | 17 | 1.67% |
| | | | | clindamycin | 212 | 58 | 5.69% | isoniazid | 34 | 9 | 0.88% |
| | | | | enoxaparin | 276 | 45 | 4.41% | infliximab | 14 | 7 | 0.69% |
| | | | | esomeprazole | 308 | 35 | 3.43% | pyrazinamide | 8 | 4 | 0.39% |
| | | | | dexamethasone | 244 | 32 | 3.14% | ethambutol | 6 | 2 | 0.20% |
| | | | | ranitidine | 240 | 25 | 2.45% | ustekinumab | 6 | 2 | 0.20% |
| | | | | fluconazole | 210 | 23 | 2.25% | dalbavancin | 2 | 1 | 0.10% |
| | | | | gentamicin | 210 | 22 | 2.16% | apremilast | 4 | 1 | 0.10% |
| | | | | meropenem | 222 | 21 | 2.06% | permethrin | 4 | 1 | 0.10% |
| | | | | ondansetron | 226 | 18 | 1.76% | acitretin | 6 | 1 | 0.10% |
| | | | | cefotaxime | 140 | 17 | 1.67% | | | | |
| | | | | cefepime | 126 | 17 | 1.67% | | | | |
| | | | | heparin | 232 | 17 | 1.67% | | | | |
| | | | | amikacin | 96 | 17 | 1.67% | | | | |
| | | | | doxycycline | 26 | 16 | 1.57% | | | | |
| | | | | flucloxacillin | 140 | 16 | 1.57% | | | | |
| | | | | cefuroxime | 148 | 15 | 1.47% | | | | |
| | | | | amiodarone | 134 | 15 | 1.47% | | | | |
| | | | | hydrocortisone | 238 | 14 | 1.37% | | | | |

**Supplementary Table S3. (continued)** Summary of the top 20 drugs of each cluster found with the Louvain algorithm in the network analysis among male ICSRs (N=1020).

| Cluster 7 ($N_{drugs}$=2) | | | | Cluster 8 ($N_{drugs}$=82) | | | |
|---|---|---|---|---|---|---|---|
| Drug | Degree | Count | Prevalence | Drug | Degree | Count | Prevalence |
| cefprozil | 2 | 1 | 0.10% | methylprednisolone | 168 | 24 | 2.35% |
| famciclovir | 2 | 1 | 0.10% | prednisone | 100 | 22 | 2.16% |
| | | | | tamsulosin | 216 | 19 | 1.86% |
| | | | | hydroxychloroquine | 98 | 17 | 1.67% |
| | | | | calcium carbonate | 192 | 14 | 1.37% |
| | | | | azathioprine | 36 | 11 | 1.08% |
| | | | | glucose | 80 | 9 | 0.88% |
| | | | | famotidine | 118 | 8 | 0.78% |
| | | | | ritonavir | 64 | 8 | 0.78% |
| | | | | hydroxyzine | 58 | 8 | 0.78% |
| | | | | itraconazole | 38 | 7 | 0.69% |
| | | | | darunavir | 58 | 7 | 0.69% |
| | | | | magnesium oxide | 90 | 6 | 0.59% |
| | | | | emtricitabine | 26 | 6 | 0.59% |
| | | | | cisplatin | 50 | 6 | 0.59% |
| | | | | nifedipine | 58 | 6 | 0.59% |
| | | | | dutasteride | 80 | 5 | 0.49% |
| | | | | mirtazapine | 66 | 5 | 0.49% |
| | | | | trimebutine | 34 | 4 | 0.39% |
| | | | | fluorouracil | 42 | 4 | 0.39% |

**Supplementary Table S3. (continued)** Summary of the top 20 drugs of each cluster found with the Louvain algorithm in the network analysis among male ICSRs (N=1020).

| **Cluster 9** ($N_{drugs}$=74) | | | | **Cluster 10** ($N_{drugs}$=3) | | | |
|---|---|---|---|---|---|---|---|
| Drug | Degree | Count | Prevalence | Drug | Degree | Count | Prevalence |
| ceftriaxone | 292 | 83 | 8.14% | ivermectin | 4 | 1 | 0.10% |
| piperacillin | 312 | 70 | 6.86% | benzyl benzoate | 4 | 1 | 0.10% |
| sulfamethoxazole | 264 | 57 | 5.59% | darbepoetin alfa | 4 | 1 | 0.10% |
| metronidazole | 284 | 56 | 5.49% | | | | |
| ciprofloxacin | 224 | 48 | 4.71% | | | | |
| azithromycin | 100 | 23 | 2.25% | | | | |
| phenytoin | 70 | 21 | 2.06% | | | | |
| valaciclovir | 78 | 17 | 1.67% | | | | |
| aciclovir | 106 | 17 | 1.67% | | | | |
| ofloxacin | 102 | 16 | 1.57% | | | | |
| levetiracetam | 154 | 16 | 1.57% | | | | |
| valproic acid | 136 | 15 | 1.47% | | | | |
| carbamazepine | 64 | 13 | 1.27% | | | | |
| mupirocin | 22 | 11 | 1.08% | | | | |
| spiramycin | 56 | 10 | 0.98% | | | | |
| phenoxymethylpenicillin | 32 | 9 | 0.88% | | | | |
| tazobactam | 42 | 8 | 0.78% | | | | |
| lamotrigine | 118 | 8 | 0.78% | | | | |
| lorazepam | 42 | 7 | 0.69% | | | | |
| temozolomide | 12 | 6 | 0.59% | | | | |

**Supplementary Table S4.** Summary of the top 20 drugs of each cluster found with the Louvain algorithm in the network analysis among female ICSRs (N=1571).

| Cluster 1 ($N_{drugs}$=157) | | | | Cluster 2 ($N_{drugs}$=86) | | | | Cluster 3 ($N_{drugs}$=10) | | | |
|---|---|---|---|---|---|---|---|---|---|---|---|
| Drug | Degree | Count | Prevalence | Drug | Degree | Count | Prevalence | Drug | Degree | Count | Prevalence |
| amoxicillin | 628 | 354 | 22.53% | ceftriaxone | 336 | 142 | 9.04% | rifampicin | 72 | 17 | 1.08% |
| furosemide | 580 | 127 | 8.08% | vancomycin | 284 | 118 | 7.51% | isoniazid | 70 | 10 | 0.64% |
| pantoprazole | 458 | 81 | 5.16% | clindamycin | 358 | 113 | 7.19% | moxifloxacin | 58 | 8 | 0.51% |
| bisoprolol | 316 | 62 | 3.95% | metronidazole | 294 | 92 | 5.86% | pyrazinamide | 22 | 6 | 0.38% |
| allopurinol | 284 | 46 | 2.93% | piperacillin | 250 | 83 | 5.28% | ethambutol | 48 | 6 | 0.38% |
| clarithromycin | 126 | 35 | 2.23% | ciprofloxacin | 194 | 64 | 4.07% | streptomycin | 14 | 1 | 0.06% |
| salbutamol | 308 | 33 | 2.10% | sulfamethoxazole | 246 | 46 | 2.93% | cycloserine | 14 | 1 | 0.06% |
| betamethasone | 220 | 28 | 1.78% | levofloxacin | 202 | 46 | 2.93% | protionamide | 4 | 1 | 0.06% |
| fluindione | 176 | 27 | 1.72% | fluconazole | 198 | 32 | 2.04% | ethionamide | 14 | 1 | 0.06% |
| azithromycin | 142 | 25 | 1.59% | ampicillin | 120 | 30 | 1.91% | tegafur | 8 | 1 | 0.06% |
| spironolactone | 220 | 24 | 1.53% | meropenem | 122 | 27 | 1.72% | | | | |
| warfarin | 166 | 21 | 1.34% | ranitidine | 202 | 21 | 1.34% | | | | |
| candesartan | 180 | 21 | 1.34% | valaciclovir | 128 | 21 | 1.34% | | | | |
| cetirizine | 208 | 19 | 1.21% | cefepime | 110 | 20 | 1.27% | | | | |
| fluticasone | 216 | 18 | 1.15% | gentamicin | 106 | 20 | 1.27% | | | | |
| oxazepam | 228 | 18 | 1.15% | cloxacillin | 98 | 19 | 1.21% | | | | |
| digoxin | 124 | 17 | 1.08% | teicoplanin | 66 | 17 | 1.08% | | | | |
| bromazepam | 208 | 17 | 1.08% | amikacin | 124 | 17 | 1.08% | | | | |
| budesonide | 182 | 16 | 1.02% | amiodarone | 168 | 16 | 1.02% | | | | |
| irbesartan | 166 | 16 | 1.02% | aciclovir | 102 | 16 | 1.02% | | | | |

**Supplementary Table S4.(continued)** Summary of the top 20 drugs of each cluster found with the Louvain algorithm in the network analysis among female ICSRs (N=1571).

| **Cluster 4** ($N_{drugs}$=1) | | | | **Cluster 5** ($N_{drugs}$=144) | | | | **Cluster 6** ($N_{drugs}$=2) | | | |
|---|---|---|---|---|---|---|---|---|---|---|---|
| Drug | Degree | Count | Prevalence | Drug | Degree | Count | Prevalence | Drug | Degree | Count | Prevalence |
| peginterferon alfa-2a | 4 | 1 | 0.06% | acetylsalicylic acid | 528 | 108 | 6.87% | anakinra | 2 | 1 | 0.06% |
| | | | | amlodipine | 404 | 75 | 4.77% | canakinumab | 2 | 1 | 0.06% |
| | | | | omeprazole | 436 | 72 | 4.58% | | | | |
| | | | | terbinafine | 242 | 63 | 4.01% | | | | |
| | | | | metformin | 384 | 62 | 3.95% | | | | |
| | | | | atorvastatin | 390 | 56 | 3.56% | | | | |
| | | | | hydrochlorothiazide | 300 | 46 | 2.93% | | | | |
| | | | | simvastatin | 274 | 41 | 2.61% | | | | |
| | | | | folic acid | 316 | 40 | 2.55% | | | | |
| | | | | diltiazem | 244 | 40 | 2.55% | | | | |
| | | | | alprazolam | 340 | 36 | 2.29% | | | | |
| | | | | ramipril | 238 | 33 | 2.10% | | | | |
| | | | | clopidogrel | 232 | 32 | 2.04% | | | | |
| | | | | pregabalin | 288 | 31 | 1.97% | | | | |
| | | | | heparin | 294 | 30 | 1.91% | | | | |
| | | | | zopiclone | 270 | 29 | 1.85% | | | | |
| | | | | enalapril | 144 | 23 | 1.46% | | | | |
| | | | | colecalciferol | 248 | 21 | 1.34% | | | | |
| | | | | lorazepam | 226 | 20 | 1.27% | | | | |
| | | | | metoprolol | 216 | 18 | 1.15% | | | | |

**Supplementary Table S4.(continued)** Summary of the top 20 drugs of each cluster found with the Louvain algorithm in the network analysis among female ICSRs (N=1571).

| Cluster 7 ($N_{drugs}$=130) | | | | Cluster 8 ($N_{drugs}$=33) | | | |
|---|---|---|---|---|---|---|---|
| Drug | Degree | Count | Prevalence | Drug | Degree | Count | Prevalence |
| paracetamol | 736 | 220 | 14.00% | levetiracetam | 168 | 52 | 3.31% |
| pristinamycin | 272 | 82 | 5.22% | valproic acid | 154 | 48 | 3.06% |
| esomeprazole | 416 | 80 | 5.09% | atenolol | 176 | 21 | 1.34% |
| enoxaparin | 376 | 72 | 4.58% | alendronic acid | 156 | 19 | 1.21% |
| tramadol | 314 | 62 | 3.95% | phenytoin | 44 | 12 | 0.76% |
| ibuprofen | 194 | 50 | 3.18% | fexofenadine | 90 | 10 | 0.64% |
| diclofenac | 216 | 34 | 2.16% | dextropropoxyphene | 86 | 8 | 0.51% |
| cefazolin | 238 | 31 | 1.97% | clonazepam | 98 | 8 | 0.51% |
| nefopam | 240 | 28 | 1.78% | thiamazole | 34 | 6 | 0.38% |
| hydroxyzine | 222 | 28 | 1.78% | pioglitazone | 30 | 4 | 0.25% |
| ketoprofen | 166 | 26 | 1.65% | clobazam | 50 | 4 | 0.25% |
| metoclopramide | 180 | 19 | 1.21% | lamivudine | 24 | 3 | 0.19% |
| phloroglucinol | 136 | 18 | 1.15% | vemurafenib | 24 | 3 | 0.19% |
| oxycodone | 198 | 18 | 1.15% | phenobarbital | 24 | 3 | 0.19% |
| morphine | 198 | 17 | 1.08% | ritonavir | 20 | 2 | 0.13% |
| propofol | 136 | 15 | 0.95% | darunavir | 20 | 2 | 0.13% |
| iomeprol | 96 | 15 | 0.95% | diflucortolone | 14 | 2 | 0.13% |
| fenofibrate | 126 | 14 | 0.89% | carbimazole | 22 | 2 | 0.13% |
| iodixanol | 92 | 14 | 0.89% | oxygen | 18 | 2 | 0.13% |
| ondansetron | 154 | 13 | 0.83% | nateglinide | 8 | 1 | 0.06% |

**Supplementary Table S4.(continued)** Summary of the top 20 drugs of each cluster found with the Louvain algorithm in the network analysis among female ICSRs (N=1571).

| **Cluster 9** ($N_{drugs}$=92) | | | | **Cluster 10** ($N_{drugs}$=86) | | | |
|---|---|---|---|---|---|---|---|
| Drug | Degree | Count | Prevalence | Drug | Degree | Count | Prevalence |
| hydroxychloroquine | 248 | 81 | 5.16% | dexamethasone | 238 | 28 | 1.78% |
| prednisolone | 280 | 77 | 4.90% | ofloxacin | 150 | 24 | 1.53% |
| prednisone | 310 | 53 | 3.37% | desloratadine | 234 | 19 | 1.21% |
| calcium carbonate | 362 | 46 | 2.93% | acetazolamide | 150 | 19 | 1.21% |
| lansoprazole | 254 | 35 | 2.23% | rosuvastatin | 198 | 18 | 1.15% |
| methotrexate | 188 | 33 | 2.10% | cefuroxime | 98 | 16 | 1.02% |
| methylprednisolone | 136 | 27 | 1.72% | cefalexin | 102 | 15 | 0.95% |
| hydrocortisone | 150 | 15 | 0.95% | ascorbic acid | 130 | 15 | 0.95% |
| acitretin | 110 | 13 | 0.83% | fluoxetine | 198 | 14 | 0.89% |
| ciclosporin | 84 | 12 | 0.76% | duloxetine | 124 | 14 | 0.89% |
| azathioprine | 52 | 11 | 0.70% | gabapentin | 150 | 13 | 0.83% |
| losartan | 102 | 11 | 0.70% | valsartan | 160 | 12 | 0.76% |
| calcitriol | 116 | 10 | 0.64% | zolpidem | 198 | 12 | 0.76% |
| flucloxacillin | 58 | 10 | 0.64% | citalopram | 138 | 12 | 0.76% |
| mefenamic acid | 28 | 10 | 0.64% | propranolol | 130 | 10 | 0.64% |
| tiaprofenic acid | 34 | 9 | 0.57% | bupropion | 44 | 10 | 0.64% |
| chlorphenamine | 64 | 9 | 0.57% | lamotrigine | 70 | 9 | 0.57% |
| domperidone | 120 | 8 | 0.51% | tobramycin | 164 | 8 | 0.51% |
| exemestane | 42 | 8 | 0.51% | aripiprazole | 44 | 8 | 0.51% |
| denosumab | 74 | 8 | 0.51% | nitrofurantoin | 102 | 7 | 0.45% |

**Supplementary Table S5.** Summary of the top 20 drugs of each cluster found with the Louvain algorithm in the network analysis among ICSRs aged < 65 years old (N=1379).

| Cluster 1 ($N_{drugs}$=61) | | | | Cluster 2 ($N_{drugs}$=93) | | | | Cluster 3 ($N_{drugs}$=57) | | | |
|---|---|---|---|---|---|---|---|---|---|---|---|
| Drug | Degree | Count | Prevalence | Drug | Degree | Count | Prevalence | Drug | Degree | Count | Prevalence |
| vancomycin | 236 | 104 | 7.54% | amoxicillin | 508 | 288 | 20.88% | ranitidine | 190 | 26 | 1.89% |
| ceftriaxone | 266 | 103 | 7.47% | paracetamol | 610 | 232 | 16.82% | clarithromycin | 132 | 23 | 1.67% |
| clindamycin | 324 | 94 | 6.82% | ibuprofen | 226 | 80 | 5.80% | flucloxacillin | 140 | 18 | 1.31% |
| metronidazole | 290 | 73 | 5.29% | pristinamycin | 208 | 66 | 4.79% | heparin | 226 | 18 | 1.31% |
| piperacillin | 182 | 57 | 4.13% | erythromycin | 80 | 20 | 1.45% | rifampicin | 122 | 16 | 1.16% |
| ciprofloxacin | 200 | 47 | 3.41% | betamethasone | 102 | 19 | 1.38% | hydrocortisone | 186 | 15 | 1.09% |
| ampicillin | 80 | 33 | 2.39% | mefenamic acid | 34 | 15 | 1.09% | cefotaxime | 124 | 15 | 1.09% |
| cefazolin | 142 | 31 | 2.25% | chlorphenamine | 54 | 15 | 1.09% | tranexamic acid | 174 | 15 | 1.09% |
| meropenem | 148 | 31 | 2.25% | fusidic acid | 98 | 12 | 0.87% | isoniazid | 46 | 12 | 0.87% |
| cloxacillin | 118 | 27 | 1.96% | cefixime | 22 | 10 | 0.73% | lactulose | 142 | 11 | 0.80% |
| gentamicin | 192 | 24 | 1.74% | tiaprofenic acid | 40 | 10 | 0.73% | chlorhexidine | 132 | 7 | 0.51% |
| doxycycline | 46 | 23 | 1.67% | cetirizine | 78 | 10 | 0.73% | minocycline | 28 | 7 | 0.51% |
| benzylpenicillin | 70 | 19 | 1.38% | naproxen | 38 | 9 | 0.65% | pyrazinamide | 22 | 7 | 0.51% |
| ceftazidime | 78 | 19 | 1.38% | acetylcysteine | 36 | 9 | 0.65% | ethambutol | 20 | 6 | 0.44% |
| amikacin | 92 | 19 | 1.38% | levocetirizine | 68 | 9 | 0.65% | clotrimazole | 34 | 5 | 0.36% |
| aciclovir | 90 | 19 | 1.38% | cefpodoxime | 42 | 8 | 0.58% | norepinephrine | 108 | 5 | 0.36% |
| ofloxacin | 72 | 13 | 0.94% | cefditoren | 14 | 8 | 0.58% | moxifloxacin | 32 | 4 | 0.29% |
| teicoplanin | 46 | 11 | 0.80% | candesartan | 66 | 8 | 0.58% | oseltamivir | 86 | 4 | 0.29% |
| linezolid | 66 | 11 | 0.80% | loratadine | 30 | 8 | 0.58% | chloramphenicol | 82 | 4 | 0.29% |
| cefoperazone | 40 | 9 | 0.65% | piroxicam | 26 | 7 | 0.51% | quetiapine | 102 | 4 | 0.29% |

**Supplementary Table S5. (continued)** Summary of the top 20 drugs of each cluster found with the Louvain algorithm in the network analysis among ICSRs aged < 65 years old (N=1379).

| **Cluster 4** ($N_{drugs}$=2) | | | | **Cluster 5** ($N_{drugs}$=10) | | | | **Cluster 6** ($N_{drugs}$=33) | | | |
|---|---|---|---|---|---|---|---|---|---|---|---|
| Drug | Degree | Count | Prevalence | Drug | Degree | Count | Prevalence | Drug | Degree | Count | Prevalence |
| cefprozil | 2 | 1 | 0.07% | ritonavir | 26 | 5 | 0.36% | allopurinol | 162 | 34 | 2.47% |
| famciclovir | 2 | 1 | 0.07% | emtricitabine | 24 | 5 | 0.36% | caffeine | 84 | 11 | 0.80% |
| | | | | cisplatin | 32 | 5 | 0.36% | carbocisteine | 82 | 11 | 0.80% |
| | | | | darunavir | 20 | 4 | 0.29% | codeine | 118 | 11 | 0.80% |
| | | | | bleomycin | 18 | 4 | 0.29% | bromhexine | 78 | 8 | 0.58% |
| | | | | etoposide | 16 | 2 | 0.15% | colchicine | 34 | 7 | 0.51% |
| | | | | cetuximab | 10 | 2 | 0.15% | ambroxol | 76 | 7 | 0.51% |
| | | | | peginterferon alfa-2a | 6 | 2 | 0.15% | tipepidine | 38 | 5 | 0.36% |
| | | | | atazanavir | 8 | 1 | 0.07% | bendamustine | 12 | 3 | 0.22% |
| | | | | capecitabine | 6 | 1 | 0.07% | pemetrexed | 42 | 3 | 0.22% |
| | | | | | | | | rituximab | 12 | 3 | 0.22% |
| | | | | | | | | acebutolol | 34 | 3 | 0.22% |
| | | | | | | | | telmisartan | 46 | 3 | 0.22% |
| | | | | | | | | etizolam | 56 | 3 | 0.22% |
| | | | | | | | | tulobuterol | 24 | 3 | 0.22% |
| | | | | | | | | theophylline | 52 | 3 | 0.22% |
| | | | | | | | | clotiazepam | 22 | 2 | 0.15% |
| | | | | | | | | palonosetron | 34 | 1 | 0.07% |
| | | | | | | | | potassium citrate | 34 | 1 | 0.07% |
| | | | | | | | | cefozopran | 4 | 1 | 0.07% |

**Supplementary Table S5. (continued)** Summary of the top 20 drugs of each cluster found with the Louvain algorithm in the network analysis among ICSRs aged < 65 years old (N=1379).

| Cluster 7 ($N_{drugs}$=2) | | | | Cluster 8 ($N_{drugs}$=49) | | | | Cluster 9 ($N_{drugs}$=82) | | | |
|---|---|---|---|---|---|---|---|---|---|---|---|
| Drug | Degree | Count | Prevalence | Drug | Degree | Count | Prevalence | Drug | Degree | Count | Prevalence |
| triptorelin | 2 | 1 | 0.07% | sulfamethoxazole | 162 | 47 | 3.41% | esomeprazole | 328 | 51 | 3.70% |
| exemestane | 2 | 1 | 0.07% | valaciclovir | 122 | 21 | 1.52% | enoxaparin | 248 | 46 | 3.34% |
| | | | | fluconazole | 120 | 20 | 1.45% | tramadol | 256 | 44 | 3.19% |
| | | | | levofloxacin | 108 | 18 | 1.31% | diclofenac | 198 | 43 | 3.12% |
| | | | | cefepime | 68 | 14 | 1.02% | dexamethasone | 226 | 32 | 2.32% |
| | | | | ciclosporin | 104 | 13 | 0.94% | ondansetron | 202 | 22 | 1.60% |
| | | | | amiodarone | 52 | 10 | 0.73% | propofol | 208 | 22 | 1.60% |
| | | | | mupirocin | 16 | 10 | 0.73% | cefuroxime | 144 | 21 | 1.52% |
| | | | | fluindione | 46 | 7 | 0.51% | ketoprofen | 116 | 21 | 1.52% |
| | | | | temozolomide | 12 | 6 | 0.44% | hydroxyzine | 154 | 21 | 1.52% |
| | | | | lamivudine | 20 | 5 | 0.36% | nefopam | 162 | 20 | 1.45% |
| | | | | mycophenolic acid | 40 | 5 | 0.36% | metoclopramide | 172 | 18 | 1.31% |
| | | | | nystatin | 36 | 4 | 0.29% | morphine | 168 | 17 | 1.23% |
| | | | | racecadotril | 36 | 4 | 0.29% | phloroglucinol | 92 | 13 | 0.94% |
| | | | | ursodeoxycholic acid | 20 | 3 | 0.22% | sufentanil | 88 | 12 | 0.87% |
| | | | | rifaximin | 12 | 3 | 0.22% | lidocaine | 126 | 11 | 0.80% |
| | | | | diosmectite | 26 | 3 | 0.22% | fentanyl | 208 | 11 | 0.80% |
| | | | | nevirapine | 6 | 3 | 0.22% | midazolam | 150 | 10 | 0.73% |
| | | | | gemcitabine | 16 | 3 | 0.22% | diphenhydramine | 114 | 9 | 0.65% |
| | | | | doxorubicin | 24 | 3 | 0.22% | rivaroxaban | 42 | 7 | 0.51% |

**Supplementary Table S5. (continued)** Summary of the top 20 drugs of each cluster found with the Louvain algorithm in the network analysis among ICSRs aged < 65 years old (N=1379).

| Cluster 10 ($N_{drugs}$=117) | | | | Cluster 11 ($N_{drugs}$=46) | | | |
|---|---|---|---|---|---|---|---|
| Drug | Degree | Count | Prevalence | Drug | Degree | Count | Prevalence |
| omeprazole | 368 | 55 | 3.99% | levetiracetam | 176 | 22 | 1.60% |
| acetylsalicylic acid | 280 | 50 | 3.63% | valproic acid | 130 | 21 | 1.52% |
| furosemide | 262 | 47 | 3.41% | phenytoin | 50 | 18 | 1.31% |
| metformin | 258 | 43 | 3.12% | carbamazepine | 68 | 17 | 1.23% |
| amlodipine | 196 | 31 | 2.25% | lorazepam | 132 | 14 | 1.02% |
| atorvastatin | 242 | 27 | 1.96% | ascorbic acid | 66 | 13 | 0.94% |
| diltiazem | 152 | 25 | 1.81% | lamotrigine | 114 | 13 | 0.94% |
| bisoprolol | 152 | 24 | 1.74% | iomeprol | 58 | 11 | 0.80% |
| simvastatin | 176 | 24 | 1.74% | bupropion | 50 | 10 | 0.73% |
| hydrochlorothiazide | 150 | 18 | 1.31% | duloxetine | 80 | 10 | 0.73% |
| perindopril | 150 | 15 | 1.09% | iodixanol | 40 | 10 | 0.73% |
| ramipril | 86 | 13 | 0.94% | famotidine | 82 | 9 | 0.65% |
| clopidogrel | 136 | 12 | 0.87% | aripiprazole | 40 | 8 | 0.58% |
| enalapril | 76 | 12 | 0.87% | clonazepam | 40 | 6 | 0.44% |
| zopiclone | 144 | 12 | 0.87% | phenobarbital | 32 | 5 | 0.36% |
| itraconazole | 32 | 10 | 0.73% | cyanocobalamin | 38 | 4 | 0.29% |
| salbutamol | 102 | 10 | 0.73% | dapsone | 20 | 4 | 0.29% |
| spironolactone | 72 | 9 | 0.65% | glucosamine | 22 | 4 | 0.29% |
| fluticasone | 116 | 9 | 0.65% | lithium | 34 | 4 | 0.29% |
| olanzapine | 60 | 9 | 0.65% | clobazam | 60 | 4 | 0.29% |

**Supplementary Table S5. (continued)** Summary of the top 20 drugs of each cluster found with the Louvain algorithm in the network analysis among ICSRs aged < 65 years old (N=1379).

| Cluster 12 ($N_{drugs}$=47) | | | | Cluster 13 ($N_{drugs}$=101) | | | |
|---|---|---|---|---|---|---|---|
| Drug | Degree | Count | Prevalence | Drug | Degree | Count | Prevalence |
| cefalexin | 104 | 15 | 1.09% | prednisolone | 260 | 78 | 5.66% |
| desloratadine | 170 | 13 | 0.94% | hydroxychloroquine | 170 | 62 | 4.50% |
| gabapentin | 140 | 12 | 0.87% | pantoprazole | 268 | 48 | 3.48% |
| fluoxetine | 116 | 11 | 0.80% | prednisone | 216 | 48 | 3.48% |
| rosuvastatin | 110 | 10 | 0.73% | terbinafine | 152 | 47 | 3.41% |
| propranolol | 88 | 9 | 0.65% | methylprednisolone | 160 | 34 | 2.47% |
| iohexol | 60 | 8 | 0.58% | methotrexate | 140 | 32 | 2.32% |
| metoprolol | 86 | 7 | 0.51% | calcium carbonate | 196 | 23 | 1.67% |
| ketoconazole | 82 | 7 | 0.51% | folic acid | 176 | 23 | 1.67% |
| ethinylestradiol | 26 | 7 | 0.51% | azathioprine | 72 | 19 | 1.38% |
| citalopram | 108 | 6 | 0.44% | azithromycin | 60 | 17 | 1.23% |
| risperidone | 54 | 5 | 0.36% | alprazolam | 168 | 15 | 1.09% |
| zolpidem | 102 | 5 | 0.36% | atenolol | 116 | 14 | 1.02% |
| sertraline | 70 | 5 | 0.36% | pregabalin | 138 | 14 | 1.02% |
| valsartan | 62 | 4 | 0.29% | celecoxib | 90 | 12 | 0.87% |
| mometasone | 56 | 4 | 0.29% | lansoprazole | 78 | 11 | 0.80% |
| bromazepam | 62 | 4 | 0.29% | domperidone | 114 | 11 | 0.80% |
| magnesium oxide | 40 | 3 | 0.22% | alendronic acid | 84 | 10 | 0.73% |
| cefaclor | 6 | 3 | 0.22% | oxycodone | 104 | 10 | 0.73% |
| tobramycin | 92 | 3 | 0.22% | fexofenadine | 64 | 10 | 0.73% |

**Supplementary Table S6.** Summary of the top 20 drugs of each cluster found with the Louvain algorithm in the network analysis for ≥ **65 years old** (N=1043).

| **Cluster 1** ($N_{drugs}$=120) | | | | **Cluster 2** ($N_{drugs}$=152) | | | | **Cluster 3** ($N_{drugs}$=68) | | | |
|---|---|---|---|---|---|---|---|---|---|---|---|
| Drug | Degree | Count | Prevalence | Drug | Degree | Count | Prevalence | Drug | Degree | Count | Prevalence |
| amoxicillin | 536 | 229 | 21.96% | acetylsalicylic acid | 554 | 120 | 11.51% | valproic acid | 136 | 41 | 3.93% |
| furosemide | 590 | 130 | 12.46% | amlodipine | 416 | 79 | 7.57% | levetiracetam | 126 | 41 | 3.93% |
| bisoprolol | 416 | 79 | 7.57% | atorvastatin | 378 | 61 | 5.85% | lansoprazole | 222 | 30 | 2.88% |
| esomeprazole | 392 | 58 | 5.56% | metformin | 320 | 45 | 4.31% | betamethasone | 208 | 22 | 2.11% |
| allopurinol | 360 | 54 | 5.18% | terbinafine | 186 | 34 | 3.26% | diclofenac | 138 | 19 | 1.82% |
| pristinamycin | 234 | 48 | 4.60% | clopidogrel | 264 | 33 | 3.16% | tamsulosin | 194 | 16 | 1.53% |
| ramipril | 292 | 41 | 3.93% | hydrochlorothiazide | 218 | 33 | 3.16% | methylprednisolone | 132 | 15 | 1.44% |
| fluindione | 182 | 30 | 2.88% | diltiazem | 220 | 33 | 3.16% | hydrocortisone | 146 | 12 | 1.15% |
| warfarin | 232 | 28 | 2.68% | metoprolol | 200 | 23 | 2.21% | famotidine | 130 | 12 | 1.15% |
| candesartan | 186 | 21 | 2.01% | pregabalin | 260 | 23 | 2.21% | magnesium oxide | 158 | 11 | 1.05% |
| spironolactone | 202 | 20 | 1.92% | cefazolin | 174 | 22 | 2.11% | carbocisteine | 128 | 11 | 1.05% |
| clarithromycin | 116 | 19 | 1.82% | glyceryl trinitrate | 238 | 22 | 2.11% | ascorbic acid | 126 | 9 | 0.86% |
| oxazepam | 208 | 19 | 1.82% | rosuvastatin | 168 | 20 | 1.92% | nifedipine | 72 | 8 | 0.77% |
| zopiclone | 194 | 19 | 1.82% | fluticasone | 196 | 17 | 1.63% | fentanyl | 92 | 8 | 0.77% |
| pravastatin | 150 | 17 | 1.63% | gliclazide | 154 | 16 | 1.53% | carbamazepine | 80 | 8 | 0.77% |
| digoxin | 156 | 16 | 1.53% | sitagliptin | 188 | 16 | 1.53% | phenytoin | 38 | 7 | 0.67% |
| torasemide | 180 | 16 | 1.53% | lisinopril | 116 | 16 | 1.53% | donepezil | 82 | 7 | 0.67% |
| insulin glargine | 184 | 15 | 1.44% | valsartan | 154 | 16 | 1.53% | propranolol | 70 | 5 | 0.48% |
| cetirizine | 200 | 15 | 1.44% | atenolol | 114 | 15 | 1.44% | finasteride | 50 | 5 | 0.48% |
| verapamil | 112 | 14 | 1.34% | escitalopram | 132 | 14 | 1.34% | ursodeoxycholic acid | 48 | 4 | 0.38% |

**Supplementary Table S6. (continued)** Summary of the top 20 drugs of each cluster found with the Louvain algorithm in the network analysis for **≥ 65 years old** (N=1043).

| **Cluster 4** (Ndrugs=2) | | | | **Cluster 5** (Ndrugs=37) | | | | **Cluster 6** (Ndrugs=51) | | | |
|---|---|---|---|---|---|---|---|---|---|---|---|
| Drug | Degree | Count | Prevalence | Drug | Degree | Count | Prevalence | Drug | Degree | Count | Prevalence |
| emtricitabine | 6 | 1 | 0.10% | paracetamol | 584 | 129 | 12.37% | hydroxychloroquine | 150 | 29 | 2.78% |
| efavirenz | 6 | 1 | 0.10% | omeprazole | 410 | 71 | 6.81% | folic acid | 278 | 29 | 2.78% |
| | | | | pantoprazole | 416 | 65 | 6.23% | prednisone | 192 | 25 | 2.40% |
| | | | | enoxaparin | 338 | 58 | 5.56% | colecalciferol | 232 | 19 | 1.82% |
| | | | | fluconazole | 188 | 30 | 2.88% | methotrexate | 94 | 14 | 1.34% |
| | | | | alprazolam | 296 | 26 | 2.49% | apixaban | 172 | 14 | 1.34% |
| | | | | heparin | 236 | 25 | 2.40% | aciclovir | 108 | 13 | 1.25% |
| | | | | dexamethasone | 172 | 23 | 2.21% | lorazepam | 154 | 13 | 1.25% |
| | | | | cefepime | 130 | 22 | 2.11% | mirtazapine | 102 | 10 | 0.96% |
| | | | | amiodarone | 198 | 20 | 1.92% | venlafaxine | 114 | 10 | 0.96% |
| | | | | meropenem | 180 | 17 | 1.63% | isoniazid | 58 | 7 | 0.67% |
| | | | | ranitidine | 174 | 16 | 1.53% | telmisartan | 62 | 5 | 0.48% |
| | | | | morphine | 156 | 13 | 1.25% | denosumab | 48 | 5 | 0.48% |
| | | | | zolpidem | 176 | 12 | 1.15% | cefdinir | 24 | 4 | 0.38% |
| | | | | lidocaine | 118 | 9 | 0.86% | ritonavir | 48 | 4 | 0.38% |
| | | | | daptomycin | 96 | 8 | 0.77% | darunavir | 48 | 4 | 0.38% |
| | | | | diphenhydramine | 70 | 8 | 0.77% | sotalol | 50 | 4 | 0.38% |
| | | | | metoclopramide | 66 | 7 | 0.67% | levocetirizine | 54 | 4 | 0.38% |
| | | | | promethazine | 92 | 7 | 0.67% | bilastine | 60 | 4 | 0.38% |
| | | | | meloxicam | 62 | 7 | 0.67% | triamcinolone | 24 | 3 | 0.29% |

**Supplementary Table S6. (continued)** Summary of the top 20 drugs of each cluster found with the Louvain algorithm in the network analysis for ≥ **65 years old** (N=1043).

| **Cluster 7** ($N_{drugs}$=3) | | | | **Cluster 8** ($N_{drugs}$=86) | | | | **Cluster 9** ($N_{drugs}$=88) | | | |
|---|---|---|---|---|---|---|---|---|---|---|---|
| Drug | Degree | Count | Prevalence | Drug | Degree | Count | Prevalence | Drug | Degree | Count | Prevalence |
| ivermectin | 4 | 1 | 0.10% | simvastatin | 318 | 39 | 3.74% | ceftriaxone | 324 | 109 | 10.45% |
| benzyl benzoate | 4 | 1 | 0.10% | prednisolone | 260 | 36 | 3.45% | vancomycin | 266 | 97 | 9.30% |
| darbepoetin alfa | 4 | 1 | 0.10% | calcium carbonate | 300 | 33 | 3.16% | piperacillin | 304 | 79 | 7.57% |
| | | | | salbutamol | 256 | 28 | 2.68% | clindamycin | 232 | 69 | 6.62% |
| | | | | budesonide | 230 | 22 | 2.11% | ciprofloxacin | 204 | 63 | 6.04% |
| | | | | lercanidipine | 152 | 16 | 1.53% | metronidazole | 252 | 61 | 5.85% |
| | | | | enalapril | 128 | 16 | 1.53% | levofloxacin | 242 | 50 | 4.79% |
| | | | | ibuprofen | 136 | 15 | 1.44% | sulfamethoxazole | 264 | 44 | 4.22% |
| | | | | alendronic acid | 136 | 14 | 1.34% | tramadol | 218 | 39 | 3.74% |
| | | | | hydroxyzine | 122 | 13 | 1.25% | azithromycin | 144 | 27 | 2.59% |
| | | | | codeine | 150 | 13 | 1.25% | ofloxacin | 142 | 25 | 2.40% |
| | | | | iodixanol | 98 | 12 | 1.15% | ampicillin | 102 | 17 | 1.63% |
| | | | | iomeprol | 90 | 12 | 1.15% | teicoplanin | 108 | 16 | 1.53% |
| | | | | nicardipine | 118 | 10 | 0.96% | rifampicin | 70 | 16 | 1.53% |
| | | | | ketoprofen | 86 | 10 | 0.96% | spiramycin | 64 | 14 | 1.34% |
| | | | | gabapentin | 62 | 10 | 0.96% | gentamicin | 124 | 14 | 1.34% |
| | | | | montelukast | 116 | 9 | 0.86% | cloxacillin | 80 | 13 | 1.25% |
| | | | | citalopram | 76 | 8 | 0.77% | cefotaxime | 78 | 10 | 0.96% |
| | | | | flucloxacillin | 52 | 7 | 0.67% | linezolid | 54 | 10 | 0.96% |
| | | | | cyanocobalamin | 114 | 7 | 0.67% | amikacin | 86 | 10 | 0.96% |

**Supplementary Table S7.** Summary of the top 20 drugs of each cluster found by using the leading eigenvector clustering to the network analysis (N=2649).

| **Cluster 1** ($N_{drugs}$=412) | | | | **Cluster 2** ($N_{drugs}$=2) | | | |
|---|---|---|---|---|---|---|---|
| Drug | Degree | Count | Prevalence | Drug | Degree | Count | Prevalence |
| paracetamol | 916 | 397 | 14.99% | cefprozil | 2 | 1 | 0.04% |
| ceftriaxone | 472 | 234 | 8.83% | famciclovir | 2 | 1 | 0.04% |
| vancomycin | 392 | 219 | 8.27% | | | | |
| clindamycin | 468 | 181 | 6.83% | | | | |
| piperacillin | 428 | 157 | 5.93% | | | | |
| metronidazole | 448 | 150 | 5.66% | | | | |
| omeprazole | 614 | 137 | 5.17% | | | | |
| enoxaparin | 484 | 118 | 4.45% | | | | |
| esomeprazole | 566 | 116 | 4.38% | | | | |
| ciprofloxacin | 358 | 116 | 4.38% | | | | |
| sulfamethoxazole | 422 | 106 | 4.00% | | | | |
| ibuprofen | 324 | 102 | 3.85% | | | | |
| tramadol | 380 | 93 | 3.51% | | | | |
| levofloxacin | 300 | 72 | 2.72% | | | | |
| levetiracetam | 270 | 71 | 2.68% | | | | |
| valproic acid | 254 | 67 | 2.53% | | | | |
| diclofenac | 296 | 64 | 2.42% | | | | |
| dexamethasone | 366 | 61 | 2.30% | | | | |
| cefazolin | 282 | 60 | 2.27% | | | | |
| fluconazole | 308 | 58 | 2.19% | | | | |

**Supplementary Table S7. (continued)** Summary of the top 20 drugs of each cluster found by using the leading eigenvector clustering to the network analysis (N=2649).

| **Cluster 3** ($N_{drugs}$=273) | | | | **Cluster 4** ($N_{drugs}$=181) | | | |
|---|---|---|---|---|---|---|---|
| Drug | Degree | Count | Prevalence | Drug | Degree | Count | Prevalence |
| amoxicillin | 786 | 573 | 21.63% | prednisolone | 432 | 120 | 4.53% |
| furosemide | 688 | 197 | 7.44% | hydroxychloroquine | 286 | 98 | 3.70% |
| acetylsalicylic acid | 692 | 189 | 7.13% | prednisone | 356 | 76 | 2.87% |
| amlodipine | 534 | 126 | 4.76% | calcium carbonate | 472 | 62 | 2.34% |
| pristinamycin | 370 | 124 | 4.68% | folic acid | 404 | 61 | 2.30% |
| pantoprazole | 538 | 121 | 4.57% | methotrexate | 222 | 50 | 1.89% |
| bisoprolol | 466 | 108 | 4.08% | alprazolam | 398 | 46 | 1.74% |
| atorvastatin | 498 | 99 | 3.74% | pregabalin | 358 | 44 | 1.66% |
| metformin | 464 | 92 | 3.47% | metoprolol | 278 | 33 | 1.25% |
| allopurinol | 450 | 92 | 3.47% | iomeprol | 146 | 30 | 1.13% |
| terbinafine | 294 | 87 | 3.28% | oxycodone | 294 | 28 | 1.06% |
| simvastatin | 406 | 65 | 2.45% | lorazepam | 262 | 28 | 1.06% |
| diltiazem | 306 | 62 | 2.34% | colecalciferol | 302 | 27 | 1.02% |
| hydrochlorothiazide | 354 | 59 | 2.23% | gabapentin | 222 | 27 | 1.02% |
| ramipril | 324 | 58 | 2.19% | budesonide | 276 | 26 | 0.98% |
| lansoprazole | 328 | 53 | 2.00% | alendronic acid | 186 | 25 | 0.94% |
| clopidogrel | 330 | 47 | 1.77% | ascorbic acid | 232 | 24 | 0.91% |
| betamethasone | 280 | 45 | 1.70% | azathioprine | 80 | 23 | 0.87% |
| salbutamol | 338 | 42 | 1.59% | naproxen | 120 | 23 | 0.87% |
| fluindione | 208 | 38 | 1.43% | glyceryl trinitrate | 238 | 22 | 0.83% |
